# Non-Destructive Testing and Evaluation of Hybrid and Advanced Structures: A Comprehensive Review of Methods, Applications, and Emerging Trends

**DOI:** 10.3390/s25123635

**Published:** 2025-06-10

**Authors:** Farima Abdollahi-Mamoudan, Clemente Ibarra-Castanedo, Xavier P. V. Maldague

**Affiliations:** Department of Electrical and Computer Engineering, Université Laval, 1065 Avenue de la Médecine, Québec, QC G1V 0A6, Canada; xavier.maldague@gel.ulaval.ca

**Keywords:** non-destructive testing (NDT), non-destructive evaluation (NDE), hybrid structures, structural health monitoring, damage detection methods

## Abstract

Non-destructive testing (NDT) and non-destructive evaluation (NDE) are essential tools for ensuring the structural integrity, safety, and reliability of critical systems across the aerospace, civil infrastructure, energy, and advanced manufacturing sectors. As engineered materials evolve into increasingly complex architectures such as fiber-reinforced polymers, fiber–metal laminates, sandwich composites, and functionally graded materials, traditional NDT techniques face growing limitations in sensitivity, adaptability, and diagnostic reliability. This comprehensive review presents a multi-dimensional classification of NDT/NDE methods, structured by physical principles, functional objectives, and application domains. Special attention is given to hybrid and multi-material systems, which exhibit anisotropic behavior, interfacial complexity, and heterogeneous defect mechanisms that challenge conventional inspection. Alongside established techniques like ultrasonic testing, radiography, infrared thermography, and acoustic emission, the review explores emerging modalities such as capacitive sensing, electromechanical impedance, and AI-enhanced platforms that are driving the future of intelligent diagnostics. By synthesizing insights from the recent literature, the paper evaluates comparative performance metrics (e.g., sensitivity, resolution, adaptability); highlights integration strategies for embedded monitoring and multimodal sensing systems; and addresses challenges related to environmental sensitivity, data interpretation, and standardization. The transformative role of NDE 4.0 in enabling automated, real-time, and predictive structural assessment is also discussed. This review serves as a valuable reference for researchers and practitioners developing next-generation NDT/NDE solutions for hybrid and high-performance structures.

## 1. Introduction

Non-destructive testing (NDT) and Non-destructive evaluation (NDE) are essential for ensuring the integrity, reliability, and performance of materials, components, and structures without causing damage or altering their functionality. These techniques are crucial across industries such as aerospace, civil infrastructure, nuclear energy, defense, and advanced manufacturing, helping to identify harmful flaws and prevent premature failure [1,2,3,4]. NDT/NDE methods are not only used during production to determine the quality of materials but also play a critical role in routine examination and maintenance to ensure long-term structural safety and performance [5]. Over time, inspection instruments and methods have evolved, and new techniques continue to emerge, enhancing the reliability and evaluation process of components and assemblies [6].

Various flaws may enter a material or component during production or arise during its service life due to inadequate technology, poor workmanship, fatigue, impact damage, or environmental conditions. These defects, whether surface or internal discontinuities, can significantly impact the subsequent performance of the component [7,8]. NDT and NDE are essential in identifying and monitoring these flaws. While NDT typically involves applying probing energy to reveal material properties or detect discontinuities, NDE extends this scope by quantifying defect characteristics and evaluating properties such as residual stress and structural integrity. Knowledge of these defects is crucial to prevent their detrimental effects on the component’s performance and to achieve improved or acceptable quality [9]. Therefore, reliable NDT/NDE tools are necessary not only to identify defects during production but also to detect and monitor flaw growth throughout the service life of a component, supporting both quality assurance and preventive maintenance. These methods ultimately enhance component performance and extend service life [5,10].

In addition to its established role in quality assurance, NDT provides a strategic advantage throughout the operational life of components by enabling condition-based monitoring and predictive maintenance planning. The ability to detect early signs of degradation or structural anomalies without halting operation allows for timely interventions, reducing the likelihood of unexpected failures and costly downtime. As asset management becomes increasingly data-driven, NDT results are essential for informed decision-making regarding the maintenance, repair, or decommissioning of critical infrastructure. Furthermore, the integration of advanced sensing technologies and digital tools has significantly improved the sensitivity, speed, and reliability of defect detection. These advancements not only enhance the safety and performance of engineering systems but also contribute to extending service life and optimizing long-term operational costs [5,9]. Table 1 summarizes the key advantages and limitations of non-destructive testing compared to destructive testing methods, highlighting its value in applications where the preservation of the component’s functionality and integrity is essential [9].

The history of non-destructive testing and evaluation is closely linked to the evolution of industrial safety and technological innovation. Early forms of NDT included visual and auditory inspection methods used to assess the condition of critical components such as steam boilers and railway infrastructure [11].

The discovery of X-rays by Wilhelm Röntgen in 1895 marked a pivotal moment in the development of NDT. Soon after, radiography was adopted to inspect metallic castings and welds, especially in critical applications [9]. In the following decades, techniques such as magnetic particle and liquid penetrant testing enabled the detection of surface-breaking cracks in ferromagnetic and non-porous materials, respectively [12,13,14]. The post-war period brought rapid advancements in NDT technologies, including ultrasonic testing for volumetric inspection and Eddy Current Testing for surface defects, particularly in the aerospace and rail applications [12,15,16,17].

By the late 20th century, NDT/NDE had evolved into a formal discipline, supported by international standards, certifications, and specialized academic research. The development of computerized systems in the 1980s and 1990s enabled the digitization and automation of many NDT techniques. Phased array ultrasonics, digital radiography, and infrared thermography became increasingly adopted due to their imaging capabilities and ability to handle complex geometries [11,15]. In the 21st century, the emergence of advanced materials, particularly composites and hybrid structures, has challenged conventional NDT approaches. This has led to the integration of multi-physics techniques, data fusion, and model-based inspection strategies. At the same time, the NDT/NDE landscape has been reshaped by digital transformation. The integration of digital technologies, such as artificial intelligence, the Internet of Things, and digital twins, is transforming traditional NDT under the concept of NDE 4.0 [18,19,20,21]. These advancements enable real-time diagnostics, automated monitoring, and predictive maintenance, shifting inspection strategies from periodic evaluations to intelligent, continuous asset management [16,22]. Alongside this technological transformation, manual inspection is increasingly being replaced by automated systems, allowing human operators to focus on data interpretation and engineering decisions [16,22]. These innovations are shifting NDE from periodic inspection to continuous, intelligent, and automated structural health monitoring.

Defects may originate from manufacturing flaws or emerge over time due to fatigue, impact, environmental exposure, or poor workmanship, making accurate, long-term monitoring essential for safety and structural integrity [23]. As no single method can fully address all defect scenarios, combining complementary NDT/NDE techniques remains critical. Ongoing advancements in smart sensors, data-driven analytics, and multimodal platforms are essential for ensuring the structural integrity, safety, and performance of next-generation infrastructure and systems [24].

Today, the increasing use of advanced and hybrid materials such as composites, fiber-reinforced polymers, and functionally graded structures poses new challenges for defect detection due to their anisotropic, heterogeneous, and layered nature [25,26]. Defects such as delamination, matrix cracking, and interfacial debonding often require multimodal inspection techniques for reliable diagnostics. Thermo-mechanical instabilities such as thermal buckling may significantly alter structural response in hybrid or layered assemblies, particularly under high-pressure or elevated-temperature conditions [27]. Traditional NDT methods like ultrasonic testing, infrared thermography, and acoustic emission are being refined and combined to meet these demands [6,28,29]. The choice of appropriate inspection techniques depends on multiple factors, including defect type, material properties, geometry, accessibility, and economic or operational constraints [30,31]. In some cases, single techniques may be adequate, but complex systems often necessitate multimodal strategies for comprehensive assessments [2,6]. These inspection difficulties stem from fundamental material behaviors. Anisotropy causes ultrasonic and electromagnetic wave propagation to vary with direction, resulting in mode conversion and distorted signal paths [32]. Heterogeneous and layered architectures introduce multiple internal boundaries, which scatter and attenuate signals, reducing penetration and resolution [25]. Interfaces and bonding layers, especially in composites, often fail subtly (e.g., delamination), making detection especially difficult without high-resolution or multimodal techniques [33]. Furthermore, high damping in polymeric matrices or resin-rich zones leads to signal loss, while complex geometries hinder transducer placement and repeatability [19]. These combined factors require the use of more advanced, adaptive, and often multimodal NDT approaches for accurate diagnostics in modern hybrid systems [2].

Capacitive sensing has emerged as a promising non-intrusive approach for evaluating materials and structures. Techniques such as coplanar capacitive sensors enable the non-contact, high-sensitivity detection of dielectric property variations, moisture content, and delamination through insulating materials [34,35,36,37,38]. By exploiting differences in permittivity between constituent materials, capacitive sensing enables the high-resolution imaging and monitoring of internal conditions within composites and hybrid assemblies [39,40,41,42,43]. Capacitive sensing techniques are increasingly being employed to evaluate hybrid and composite systems due to their ability to detect subsurface defects, variations in dielectric properties, and interfacial delamination without damaging the material [44,45,46,47,48]. This technique offers unique advantages in hybrid material inspection and is gaining interest as part of multimodal NDT platforms [49,50,51]. Capacitive sensing is also used in multimodal systems alongside ultrasound or electromagnetic testing, enabling AI-driven diagnostics and structural health monitoring [33,34,52].

The successful implementation of modern NDT and NDE requires not only correct technique selection but also a deep understanding of each method’s underlying principles, limitations, and application domains. This demands an interdisciplinary perspective and continued advancement of the theoretical and practical dimensions of NDT [53].

Table 2 presents key milestones in the evolution of NDT technologies over the past century, emphasizing their chronological progression and increasing complexity.

The classification of NDT and NDE techniques is fundamental to understanding their underlying principles, practical capabilities, and suitability for diverse materials and structural scenarios. While traditional reviews often organize methods based on physical interaction mechanisms such as acoustic, electromagnetic, or thermal principles [2,4,6], modern engineering applications increasingly require a broader, multi-dimensional framework. This need is particularly acute in the context of hybrid, layered, and multifunctional structures, which present complex challenges in damage detection, monitoring, and characterization [3,54,55]. These materials often exhibit anisotropic behavior, embedded interfaces, and layer-dependent damage mechanisms [25,26,54], rendering traditional single-mode techniques insufficient and necessitating refined or integrated NDT approaches. In parallel, the field of NDE is experiencing a paradigm shift driven by advancements in sensor automation, real-time data acquisition, and AI-driven diagnostics. These developments are accelerating the transition toward intelligent, continuous structural monitoring systems, especially within the frameworks of structural health monitoring and predictive maintenance [4,55]. Against this backdrop, emerging technologies such as capacitive sensing, embedded sensor networks, and data-centric NDE 4.0 paradigms are reshaping structural assessment practices and broadening the operational scope of traditional methods [21,49,56,57,58].

To address these evolving demands, this review adopts a comprehensive, multi-dimensional classification framework that organizes NDT/NDE techniques across three principal dimensions: (1) the physical principle, (2) the functional purpose, and (3) the application domain. While many existing reviews focus primarily on method types or specific material systems, this work presents an integrated perspective that bridges fundamental mechanisms with practical deployment, especially in the context of hybrid and advanced structures. Emphasis is placed on emerging sensor technologies, multimodal platforms, and AI-enhanced inspection systems, highlighting their growing role in intelligent diagnostics and real-time structural health monitoring. The organization of the paper is as follows: Section 2 categorizes techniques by physical principles, Section 3 by functional objectives, and Section 4 by domain-specific applications. Section 5 discusses emerging trends and future directions, followed by comparative evaluation and selection criteria in Section 6. Section 7 presents a detailed discussion, and Section 8 concludes with key findings and recommendations for future research.

This review aims to serve as a strategic reference for researchers, engineers, and practitioners advancing next-generation NDT/NDE technologies. It focuses specifically on non-destructive testing and evaluation techniques as applied to hybrid and advanced structural materials, including composites, fiber–metal laminates, sandwich structures, and functionally graded materials. Emphasis is placed on methods relevant to damage detection, structural health monitoring, and lifetime prediction in systems characterized by anisotropy, heterogeneity, and layered architectures. Traditional and emerging techniques are assessed based on their physical principles, functional capabilities, and application-specific performance. The classification framework adopted herein is designed to move beyond conventional taxonomies by integrating physical, functional, and domain-based perspectives, offering a more holistic understanding of NDT/NDE challenges and solutions in modern multi-material systems.

### Methodology

This review adopts a structured, expert-informed literature assessment aimed at synthesizing the state of the art in non-destructive testing (NDT) and non-destructive evaluation (NDE) techniques, with a particular emphasis on their application to hybrid and advanced structural materials. The goal is to map technological developments, emerging trends, and comparative performance within the evolving context of materials exhibiting anisotropy, multi-layered architectures, and complex interfacial behaviors.

*Literature Identification and Selection*: The literature survey was conducted using academic databases such as Scopus, Web of Science, IEEE Xplore, and Google Scholar. The search strategy focused primarily on peer-reviewed publications from 2000 onward, reflecting the acceleration of research in advanced and hybrid materials. However, seminal works from earlier decades (e.g., 1989, 1993) were also included when foundational to specific techniques or historical developments in the field.

Search terms were constructed using Boolean logic and included combinations such as (“non-destructive testing” OR “non-destructive evaluation” OR “NDT” OR “NDE”) AND (“composites” OR “hybrid structures” OR “fiber-metal laminates” OR “sandwich structures” OR “advanced materials”) AND (“damage detection” OR “structural health monitoring” OR “inspection” OR “lifetime prediction”).

The initial screening process focused on titles and abstracts to identify relevant studies. A core set of approximately 150–160 articles was then reviewed in detail. From these, a curated selection of 143 references was ultimately included in this manuscript based on their technical relevance, methodological depth, and coverage of diverse physical principles and application domains.

*Thematic Organization and Synthesis Approach*: Rather than adopting a purely systematic or bibliometric format, this review employs a multi-dimensional thematic framework to organize the selected literature. This framework classifies NDT/NDE methods along three principal dimensions: (1) physical principle—the underlying sensing mechanism (e.g., acoustic, electromagnetic, thermal); (2) functional purpose—the intended diagnostic objective (e.g., detection, monitoring, characterization); and (3) application domain—the industrial or structural context (e.g., aerospace, infrastructure, energy). This approach supports a comprehensive evaluation of the performance, limitations, and integration potential of each technique, particularly in the context of emerging challenges associated with hybrid and layered structures. Attention is given to recent innovations in multimodal platforms, embedded sensing, and AI-enhanced diagnostics, as well as to key historical milestones that have shaped the evolution of the field.

In this review paper, the thematic organization and scope were informed by observable publication trends and keyword prevalence across major databases. A preliminary co-occurrence scan of indexed literature revealed consistent clustering of terms such as “non-destructive testing,” “composites,” “structural health monitoring,” and “hybrid materials,” particularly in publications from the last 10–15 years. This trend reflects a growing emphasis on multimaterial systems and intelligent diagnostics. The review structure, organized by physical principle, functional purpose, and application domain, was therefore chosen to reflect the multidimensional challenges observed in current literature and to address the increasing integration of sensing modalities in advanced structural applications.

## 2. Classification of NDT and NDE Techniques by Physical Principle

NDT/NDE techniques can be systematically classified based on the fundamental physical principles that govern their sensing mechanisms. These principles not only define how probing energy interacts with materials but also influence key performance characteristics such as sensitivity, resolution, depth of penetration, and applicability to various material systems, including metals, fiber-reinforced polymers, sandwich composites, and fiber–metal laminates [25,59,60,61]. Based on a synthesis of the current literature, the primary physical domains include mechanical (elastic wave-based), electromagnetic, radiographic (ionizing radiation), thermal/optical, and electrical techniques. Additionally, hybrid or multiphysics approaches that combine two or more principles are gaining traction, offering enhanced diagnostic capabilities for complex, anisotropic, or layered structures [25,54,62]. This classification framework highlights not only operational differences among techniques but also their alignment with the demands of modern structural health assessment.

### 2.1. Mechanical and Elastic Wave-Based Methods

These NDT/NDE techniques rely on the propagation, reflection, or emission of mechanical waves within a material and are particularly effective for detecting internal flaws such as delamination, voids, and cracks. The most prominent among them are as follows.

*Ultrasonic Testing (UT)*: A widely used volumetric NDT method that utilizes high-frequency sound waves to detect internal defects, thickness variations, and delamination in metals and composite materials. Ultrasonic testing works by analyzing the reflection, transmission, and scattering of ultrasonic waves within the test object. Advanced techniques such as phased array ultrasonic testing (PAUT) enable beam steering and enhanced imaging for complex geometries, while time-of-flight diffraction (TOFD) offers improved resolution and accurate defect sizing, particularly in thick composite or hybrid laminates. Ultrasonic testing is extensively applied in aerospace, pipeline, and structural steel inspection [2,6,25,59,63].

Recent advancements in ultrasonic testing have led to the development of high-resolution imaging methods such as the Total Focusing Method (TFM), a post-processing technique that synthetically focuses ultrasonic data at every point in the region of interest (ROI). TFM is typically implemented via Full Matrix Capture (FMC), where each array element transmits sequentially and all elements receive, allowing for complete data collection across all transmit-receive paths [64,65,66,67]. This approach enhances image quality, improves signal-to-noise ratio (SNR), and enables accurate flaw localization and sizing. Multiple wave modes can be reconstructed to adapt to different material properties and flaw orientations [65,66]. TFM has been successfully applied to the inspection of thick and attenuating materials, complex welds, and hybrid structures. Recent developments also include Rayleigh wave TFM, enabling real-time surface and sub-surface imaging with sub-100 µm resolution, even on coated or complex geometries [68]. Post-processing enhancements such as Phase Coherence Imaging (PCI), Amplitude-Phase Product Imaging (APPI), and Delay Multiply and Sum (DMAS) further improve contrast and resolution, facilitating precise defect characterization [65,68]. These innovations make TFM a leading technique for high-fidelity ultrasonic NDE, especially in applications involving safety-critical, layered, or anisotropic materials.

Another significant advancement in ultrasonic testing is the development of laser ultrasonic testing (LUT), which enables the non-contact, high-resolution inspection of composite and metallic components. Instead of using mechanical coupling, LUT employs pulsed lasers and optical detection to inspect complex or high-temperature structures [69,70]. Recent innovations include air-coupled laser ultrasound (ACLU) systems, which achieve large-field, high-resolution detection of internal defects in metal/CFRP hybrid composites, overcoming impedance mismatches and material inhomogeneity [71]. These systems demonstrate superior signal-to-noise ratios and accuracy in identifying delaminations, disbonds, and cracks compared to traditional methods. LUT is increasingly applied in aerospace and energy sectors for the fast, precise, and non-contact inspection of safety-critical structures [70,72].

*Acoustic Emission (AE)*: Acoustic emission captures transient elastic waves generated by stress-induced events such as matrix cracking, fiber fracture, or delamination. This technique is particularly effective for the real-time monitoring of progressive damage in composite and hybrid materials, especially during load testing. It enables the assessment of active defect mechanisms as they occur, making it highly suitable for structural health monitoring under operational conditions [4,6,29,33,73].

These methods are highly sensitive to elastic discontinuities, making them well-suited for volumetric inspection of both metallic and non-metallic structures. However, their effectiveness can be limited in attenuative or multilayered materials, such as thick composites, where wave scattering and signal damping occur. In such cases, advanced signal processing or hybrid approaches may be required to maintain accuracy [3].

### 2.2. Electromagnetic Methods

Electromagnetic methods exploit interactions between electromagnetic fields and the conductive or dielectric properties of materials to detect flaws, monitor conductivity, and characterize material structure. They are particularly effective for conductive materials and show growing potential in hybrid systems with embedded conductive elements or interfaces [25,74]. Major techniques include

*Eddy Current Testing (ECT)*: This technique detects surface and near-surface defects in conductive materials through electromagnetic induction. It is particularly effective for identifying cracks, corrosion, and wear in metallic components, including metallic skins and embedded conductive layers in fiber–metal laminates. Eddy Current Testing is widely used in the aerospace and rail industries due to its high sensitivity and rapid inspection capability [2,4,6,17].

*Magnetic Particle Testing (MT)*: This method is used to detect surface and near-surface defects in ferromagnetic materials by applying a magnetic field and using magnetic particles to visualize discontinuities. It operates based on magnetic flux leakage and is particularly effective for inspecting welds and structural steels. However, its application is limited to ferromagnetic materials and is not suitable for composites or non-ferromagnetic hybrid structures [1,2,14].

*Microwave Testing*: Microwave Testing uses high-frequency signals to detect subsurface defects like delamination, voids, and moisture in non-metallic materials by analyzing dielectric property changes. Sweep-frequency microwave sensors and embedded antenna arrays are being explored for inspecting thick non-metallic layers, adhesive joints, and subsurface voids in composites and concrete [33,73]. Recent advancements include near-field imaging, machine learning for defect classification, and portable systems for field use [25,60,75].

*Capacitive Sensing*: This emerging technique operates by detecting changes in dielectric permittivity in the inspected material. Coplanar capacitive sensors are particularly well-suited for the non-contact, high-sensitivity assessment of insulating layers, dielectric materials, and complex hybrid structures. Capacitive sensing is especially effective for identifying moisture ingress, delamination, bonding defects, and material discontinuities in non-metallic systems such as composites and sandwich panels. Its effectiveness in scenarios where traditional electromagnetic methods struggle, such as non-conductive or layered materials, makes it a promising solution, particularly when integrated into multimodal or hybrid sensing platforms [34,49,52,76].

Capacitive sensing stands out in this group as one of the few electromagnetic methods effective on non-conductive and heterogeneous materials, complementing eddy current and magnetic particle methods, which are limited to conductive and ferromagnetic systems [14,17,34].

### 2.3. Thermal and Optical Methods

Thermal and optical methods detect surface or subsurface defects by monitoring heat flow or light interaction, making them particularly useful for materials with varying thermal properties, such as composites [77,78]. Common methods include the following.

*Infrared thermography (IRT)*: This technique measures surface temperature distributions to identify subsurface anomalies such as delaminations, disbonds, or voids. It can be performed in active mode using an external heat source or passively under ambient conditions. Subsurface defects disrupt heat flow, creating detectable thermal contrasts. Pulsed thermography, in particular, is effective for the large-area inspection of fiber-reinforced polymer and sandwich panels. Infrared thermography offers a non-contact, rapid inspection method widely used in aerospace and civil infrastructure applications [4,6,25,63,79].

*Shearography*: Shearography, also referred to as speckle pattern shearing interferometry, falls within the category of optical and interferometric techniques. Its core physical principle is based on laser interferometry, where deformation-induced optical phase shifts are measured across a component’s surface [80]. These phase shifts arise from localized strain variations when the structure is subjected to external loading, mechanical, thermal, vibrational, or vacuum-induced forces [74]. By capturing the interference patterns between sheared images of the laser-speckle field, shearography reveals minute surface displacement gradients, which are indicative of subsurface anomalies [81]. This strain-based optical sensing mechanism makes it highly effective for identifying defects such as disbonds, delaminations, and voids, particularly in composite and sandwich structures with complex geometries and layered configurations [82].

*Digital Image Correlation (DIC)*: This technique is classified as an optical and vision-based technique grounded in the physical principles of photogrammetry and image correlation. It involves tracking the displacement of surface speckle patterns captured through sequential high-resolution images during mechanical loading. By analyzing these displacements, DIC maps full-field surface strain distributions with high accuracy. Unlike techniques that probe material volume, digital image correlation passively observes deformation responses on the surface, making it especially effective for detecting localized strain concentrations caused by subsurface damage such as delamination or debonding. Its non-contact nature, compatibility with complex geometries, and ability to assess hybrid and composite structures under real-world loading make it a valuable tool in both experimental mechanics and structural health monitoring applications [33,83,84].

*Terahertz Imaging*: Terahertz imaging is an electromagnetic technique based on the interaction of terahertz-frequency radiation with material interfaces. It relies on variations in dielectric permittivity and refractive index to detect subsurface discontinuities such as delaminations, voids, or inclusions. As terahertz waves penetrate low-density, non-conductive materials, reflected or transmitted signals reveal internal structural variations with high spatial resolution. The technique’s physical foundation in non-ionizing wave propagation makes it particularly suitable for inspecting multilayer polymer composites and dielectric-rich systems [25,33].

Thermal and optical methods are classified based on their reliance on the physical principles of heat conduction, infrared radiation, and light–matter interaction. These techniques detect anomalies by capturing surface temperature distributions, deformation gradients, or optical phase shifts that result from subsurface defects affecting thermal flow or structural response. Their non-contact, full-field, and real-time visualization capabilities, make them especially advantageous for inspecting composites and hybrid structures, where anisotropy and complex interfaces challenge conventional point-based techniques. Due to their sensitivity to delaminations, disbonds, and voids, these methods are widely used for large-area inspections in aerospace, civil, and manufacturing applications [6,25,33].

### 2.4. Radiographic and Ionizing Radiation Methods

Radiographic and ionizing radiation methods employ penetrating radiation, such as X-rays, gamma rays, or neutrons, to visualize the internal structure of a component. Common methods include the following.

*Radiographic Testing (RT)*: This method is based on the physical principle of differential attenuation of ionizing radiation, typically X-rays or gamma rays, as it passes through a material. When high-energy photons interact with the internal structure of a component, variations in material density, thickness, and atomic number cause differences in radiation absorption. These differences are recorded as contrast variations on radiographic film or digital detectors, forming a two-dimensional projection image of the internal volume. Because denser regions or defects such as voids, porosity, inclusions, and cracks attenuate radiation differently than the surrounding material, radiographic testing provides an effective means for visualizing volumetric anomalies in welds, castings, and multi-layered structures. The technique is particularly well-suited for metallic systems and high-density composites where internal features are not accessible by surface or wave-based methods. Modern digital radiography and computed radiography enhance resolution, speed, and portability, making RT a core tool in critical sectors such as aerospace, energy, and heavy manufacturing [1,2,6,85].

*Computed Tomography (CT)*: Computed tomography is grounded in the physical principle of X-ray attenuation and tomographic reconstruction. As X-rays pass through a material, their intensity is reduced based on internal variations in density and atomic number. Computed tomography acquires multiple radiographic projections at different angles and reconstructs a three-dimensional volumetric image using computational algorithms. This enables the high-resolution visualization of internal defects such as porosity, inclusions, and delaminations. Computed tomography’s strength lies in its ability to resolve complex geometries and layered structures that are difficult to assess using conventional 2D radiography [1,2,54,59,85].

*Neutron Radiography*: This method offers superior sensitivity to low-atomic-number elements such as hydrogen, making it highly effective for detecting water ingress, delamination, and matrix cracking in polymer composites. Unlike X-rays, neutrons interact strongly with hydrogen atoms, allowing the detailed visualization of moisture and damage within composite materials. This makes NR particularly valuable in applications such as aerospace, where early-stage degradation can be critical. However, the technique requires access to specialized facilities like research reactors or spallation neutron sources, which limits its widespread use. Furthermore, neutron imaging typically involves longer exposure times and lower spatial resolution compared to advanced X-ray methods [25,33,86].

These techniques provide detailed internal imaging and are especially effective for metallic and dense materials. However, they often face challenges with inspection throughput and limited material contrast when applied to low-density, layered, or fiber-rich composite structures unless enhanced by contrast agents or dual-energy techniques [25,54,59].

### 2.5. Electrical and Impedance-Based Methods

Electrical and impedance-based methods rely on the principle of electrical signal propagation and impedance variation to detect material degradation. These techniques involve the injection or sensing of electrical currents or voltage across a structure, where changes in conductivity, resistance, or electromechanical impedance are indicative of damage such as cracking, corrosion, or debonding. For example, Electrical Resistance Tomography (ERT) and impedance spectroscopy track spatial variations in resistance to localize material changes, often using embedded sensor grids. Electromechanical impedance (EMI) utilizes piezoelectric transducers to detect localized stiffness changes through variations in electrical impedance, making it suitable for health monitoring in composite and metallic systems [87,88].

### 2.6. Hybrid and Multiphysics Techniques

Hybrid and multiphysics NDT techniques are grounded in the physical principle of cross-domain sensing, where multiple physical phenomena such as acoustic, thermal, electromagnetic, or capacitive effects are jointly analyzed to improve diagnostic accuracy. These methods aim to overcome the limitations of single techniques by leveraging complementary strengths. Multimodal sensing systems, for example, integrate ultrasonic testing with thermography, or capacitive sensing with acoustic emission, to enhance coverage across different materials and geometries while reducing false positives [33,73].

Recent developments emphasize the use of AI-supported data fusion from thermal and optical domains (e.g., infrared thermography and digital image correlation), enabling automated damage classification and anomaly detection using machine learning algorithms [33,63]. Additionally, embedded wireless platforms, including radio-frequency identification (RFID), microelectromechanical systems (MEMS), and chipless microwave antennas, are being integrated into composite and layered structures for autonomous, real-time structural health monitoring. These hybrid approaches are especially effective in multifunctional or complex architectures, such as aerospace skins, sandwich panels, or smart infrastructure, where damage is distributed or interacts across multiple material interfaces [33,73].

Table 3 presents a classification of NDT/NDE techniques based on their physical principles. This classification provides foundational insight into the operational strengths and limitations of NDT/NDE methods. This categorization is particularly valuable for selecting appropriate techniques for hybrid structures, where complex interactions between materials, interfaces, and geometry require tailored sensing strategies. Techniques such as capacitive sensing, shearography, Microwave Testing, and multimodal AI-supported platforms are increasingly vital in meeting these demands.

## 3. Classification of NDT and NDE Techniques by Functional Purpose

While the classification of NDT/NDE techniques by their underlying physical principles provides foundational insight into how they operate, an equally important and often more application-driven perspective is their classification based on functional purpose. This functional categorization groups techniques according to their practical objectives, such as defect detection, damage localization, property characterization, structural monitoring, and integrity or lifetime assessment. Such an approach provides significant value for engineers, researchers, and inspectors, particularly when dealing with complex material systems like composites and hybrid structures, where multiple damage mechanisms and interfacial phenomena may coexist. This section examines these five major functional categories, critically evaluating each in terms of suitability, limitations, and integration potential, especially in advanced structural applications that demand multifaceted inspection strategies. It covers techniques for multi-scale flaw detection, emphasizes methods for localizing and quantifying damage, and highlights approaches for characterizing mechanical, thermal, and dielectric properties. The discussion further includes strategies for continuous structural monitoring and concludes with techniques focused on integrity validation and lifetime prediction, where advanced NDE tools contribute to prognostics and long-term performance modeling.

### 3.1. Defect Detection

Defect detection represents the most fundamental purpose of NDT, aimed at identifying surface and subsurface flaws that may compromise structural integrity. These defects may originate during manufacturing, such as voids, porosity, and inclusions, or develop during service, including matrix cracking, fiber breakage, delamination, and corrosion. Detecting such anomalies is especially challenging in anisotropic or layered materials, where damage may be hidden or interact across multiple interfaces. Various NDT techniques, each with distinct strengths and limitations, are employed to detect these defects, offering solutions tailored to the specific challenges presented by different materials and damage mechanisms [25,33,54].

*Ultrasonic testing (UT)* plays a central role in NDT/NDE, particularly for the detection of internal flaws such as delaminations, voids, cracks, and inclusions. This technique operates by transmitting high-frequency acoustic waves into a material and analyzing the reflected, diffracted, or scattered signals from internal discontinuities. Ultrasonic testing is especially effective in identifying volumetric and planar defects in fiber-reinforced composites, sandwich structures, and bonded joints. Phased array ultrasonic testing (PAUT) and time-of-flight diffraction (TOFD) stand out as leading techniques in advanced ultrasonic testing, recognized for their exceptional ability to provide high-resolution flaw characterization. Phased array ultrasonic testing enables precise beam steering, focusing, and sectorial scanning, offering detailed volumetric imaging even in complex, curved, or layered geometries. Time-of-flight diffraction enhances defect detection and sizing by capturing tip diffraction signals with high sensitivity, making it particularly valuable for crack depth estimation and integrity assessment. These capabilities collectively render ultrasonic testing highly versatile for comprehensive inspection tasks where surface-based methods may fall short [4,6,25,63,90].

*Eddy Current Testing (ECT) and Magnetic Particle Testing (MT)* are widely used methods for detecting surface and near-surface defects in conductive and ferromagnetic materials, respectively. Eddy Current Testing operates on the principle of electromagnetic induction, where circulating eddy currents are induced in the material and monitored for disruptions caused by surface-breaking or subsurface flaws such as cracks, pits, corrosion, or inclusions. It is highly sensitive to shallow discontinuities and can detect minute flaws without direct contact, making it valuable for inspecting aerospace skins, fastener holes, and conductive structural components. In contrast, Magnetic Particle Testing utilizes the leakage of magnetic flux around surface defects when a magnetic field is applied to a ferromagnetic component. Flaws such as cracks or laps cause a disruption in the magnetic field, leading to flux leakage, which becomes apparent through the accumulation of magnetic particles. Magnetic Particle Testing is particularly effective for crack detection in weld zones, fasteners, and fatigue-prone steel structures. Both techniques offer fast, localized inspection with minimal surface preparation but are limited to conductive (ECT) or ferromagnetic (MT) materials. Their effectiveness is limited in multi-material assemblies or dielectric layers found in hybrid composites, sandwich structures, or fiber-reinforced polymers, restricting their use in complex, multi-layered systems [2,4,6,14,17].

*Radiographic testing (RT) and computed tomography (CT)* are widely used for detecting volumetric defects such as voids, porosity, inclusions, and internal cracks in metallic components, dense composites, and high-value structural parts. Computed tomography extends the capabilities of radiographic testing by reconstructing three-dimensional images from multiple radiographic projections, enabling the detailed localization and characterization of hidden defects, especially in complex or layered assemblies. This is particularly beneficial when defect geometry, orientation, or interactions at multi-material interfaces must be resolved. Despite their diagnostic strength, both radiographic testing and computed tomography are typically confined to controlled environments due to equipment size, cost, and safety considerations [1,2,25,85,89].

*Infrared thermography (IRT)*, particularly in pulsed and lock-in modes, is well-suited for detecting defects such as disbonds, moisture ingress, and delaminations in fiber-reinforced polymers and sandwich structures. Analyzing thermal responses following stimulation provides full-field, non-contact imaging that is efficient for large-area inspections. Infrared thermography’s sensitivity to thermal gradients makes it highly effective in quality assurance and the maintenance of layered or bonded systems, especially where other NDT methods may be limited [33,73,77,91].

*Capacitive Sensing*, especially through coplanar capacitive sensors, offers a promising non-contact approach for detecting interfacial defects such as adhesive failure and disbonds in dielectric or hybrid structures. By measuring local permittivity variations, coplanar capacitive sensors can identify subtle changes associated with material degradation. Unlike conductivity or coupling-dependent methods, capacitive sensors are well-suited for inspecting fiber-reinforced polymer with metal components, bonded joints, and flexible substrates. Their scalability and adaptability make them effective in capturing defect modes often inaccessible to conventional electromagnetic or mechanical NDT techniques. In hybrid systems involving both conductive and non-conductive layers, combining capacitive sensing with ultrasonic testing or thermography can provide more comprehensive detection coverage [17,33,49,52].

### 3.2. Damage Localization

Once a defect is detected, the precise localization of its position and extent becomes crucial for assessing its impact, guiding repairs, prioritizing load redistribution, and supporting performance prediction or prognostics. Techniques in this category focus on spatial mapping of flaw geometry or progressive damage.

*Acoustic emission (AE)* enables real-time damage localization by triangulating elastic waves emitted during service-induced degradation in structures such as bridge tendons, pressure vessels, and aerospace composites. Acoustic emission systems use sensor arrays to determine defect coordinates based on time-of-arrival differences and wave velocity. Advanced signal processing, such as wavelet transforms and machine learning, improves accuracy by filtering noise and classifying defect types, even in complex anisotropic materials like fiber-reinforced polymers. Acoustic emission’s high spatial resolution makes it valuable for detecting early-stage phenomena like micro-cracks before they evolve into critical failures. When integrated into structural health monitoring systems, acoustic emission enables automated, continuous tracking of damage over large-scale infrastructure, such as wind turbine blades or pipelines, where conventional NDT methods may fall short in sensitivity or coverage [4,25,29,33,73,92].

*Shearography and digital image correlation (DIC)* are advanced optical methods that enhance defect localization through the high-resolution visualization of surface strain fields. Their strength lies in precisely identifying stress concentrations and deformation gradients in complex materials like fiber–metal laminates and carbon-fiber-reinforced polymers. Beyond their core sensing principles, modern implementations of shearography apply phase-shifting techniques to improve measurement sensitivity and minimize environmental noise. Meanwhile, digital image correlation benefits from machine-learning-enhanced pattern recognition and sub-pixel correlation algorithms, enabling the detection of micro-scale deformation around cracks or impact sites with exceptional spatial resolution. These techniques complement each other in hybrid NDT frameworks. Shearography offers rapid detection of hidden defects through strain-induced displacement gradients, and digital image correlation enables full-field, quantitative strain mapping under load. Their integration with structural health monitoring systems supports the real-time tracking of damage initiation and propagation, especially in aerospace, automotive, and civil structures where early intervention is critical [25,33,54,63,93,94,95].

*Infrared thermography (IRT)* can serve as a powerful localization tool by visualizing spatial thermal contrast patterns that correlate with internal voids, delaminations, or moisture ingress, particularly in fiber-reinforced polymers, sandwich structures, and hybrid assemblies. When paired with AI-based thermal image segmentation, such as convolutional neural networks (CNNs) and deep learning, infrared thermography achieves enhanced spatial resolution and automated defect identification by distinguishing subtle thermal anomalies from background noise. These techniques enable sub-millimeter localization accuracy, even in anisotropic or multi-layered materials where thermal diffusion is complex. Additionally, integrating infrared thermography with complementary NDT techniques like ultrasonic testing or shearography allows for multimodal defect correlation, yielding more comprehensive damage characterization. Such advancements make infrared thermography highly effective for real-time structural health monitoring across critical sectors, including aerospace, wind energy, and civil infrastructure, where the early detection of localized damage is essential for predictive maintenance and operational safety [25,33,77,80,96].

*Microwave Testing*, especially with embedded sweep-frequency antenna arrays, offers high-resolution localization of subsurface defects such as adhesive disbonds and moisture pockets in dielectric and multi-layered fiber-reinforced polymer structures [73]. Utilizing broad-spectrum microwave signals (e.g., 1–15 GHz) enables precise spatial mapping by analyzing changes in reflected or transmitted signals due to local dielectric discontinuities. Advanced processing techniques, including synthetic aperture radar imaging and machine-learning-based anomaly detection, enhance localization accuracy, achieving sub-millimeter precision even in anisotropic materials. Integration with structural health monitoring systems, such as radio-frequency identification (RFID)-enabled microwave sensors, enables non-contact, real-time monitoring of damage evolution in aerospace, wind energy, and civil infrastructure components. Combined with complementary NDT methods like infrared thermography or ultrasonic testing, Microwave Testing supports robust, multimodal characterization of internal flaws, making it indispensable for inspecting critical composite and hybrid systems [25,33,73,97,98].

Localization in hybrid systems often requires techniques that can operate across layers with differing wave speeds or thermal diffusivities favoring multimodal methods or those enhanced with signal-processing algorithms.

### 3.3. Property Characterization

Beyond flaw detection, many NDT/NDE techniques are capable of quantifying or estimating key material properties such as stiffness, dielectric permittivity, conductivity, thermal diffusivity, and acoustic impedance. This ability to provide quantitative data on physical, mechanical, or dielectric properties is essential for supporting quality control, material verification, and performance evaluation. The accurate characterization of these properties enables the assessment of material integrity, optimization of design parameters, and prediction of the behavior of structures under different environmental or loading conditions. Furthermore, integrating property characterization with advanced modeling and simulation techniques enhances the overall reliability and precision of the testing process, contributing to more effective structural health monitoring and life-cycle management.

*Electromechanical impedance (EMI) and electrical impedance tomography (EIT)* are effective for characterizing local variations in stiffness, damping, or conductivity, often linked to degradation or environmental exposure. Electromechanical impedance offers high sensitivity to changes in local stiffness and damping, making it valuable for embedded structural health monitoring networks in fiber-reinforced polymers and metallic systems. Electromechanical impedance utilizes piezoelectric transducers to measure impedance signatures that reflect mechanical properties at the sensor–material interface, enabling the real-time assessment of localized damage, such as micro-cracks or corrosion. Shifts in resonance frequencies or impedance peaks can detect material degradation from fatigue, impact, or environmental factors like moisture ingress. Electrical impedance tomography reconstructs conductivity maps by injecting low-frequency electrical currents and measuring voltage differences, providing insights into dielectric properties and internal anomalies in conductive composites or hybrid structures. Advanced signal processing, including frequency-domain analysis for electromechanical impedance and inverse algorithms for electrical impedance tomography, enhances precision, achieving high sensitivity to anisotropic behavior in multi-layered fiber-reinforced polymers or fiber–metal laminates [6,25,88,99].

*Ultrasonic spectroscopy* measures elastic modulus and acoustic impedance, providing valuable insights into stiffness, fiber–matrix bonding, and matrix consolidation quality in composites. This technique is particularly effective for assessing fiber orientation and bonding quality in fiber-reinforced polymers and hybrid laminates [3,25]. By analyzing the frequency-dependent response of ultrasonic waves, it quantifies material properties such as Young’s modulus, shear modulus, and Poisson’s ratio, offering a non-destructive means to evaluate mechanical performance and structural integrity in multi-material systems like carbon-fiber-reinforced polymers and fiber–metal laminates. Advanced signal processing techniques, such as Fourier transforms, wavelet analysis, and machine-learning-based classification, improve property characterization by isolating variations in wave attenuation and phase shifts linked to matrix porosity, fiber misalignment, or interfacial debonding. These capabilities allow ultrasonic spectroscopy to detect early-stage degradation, such as micro-voids or weakened fiber-matrix interfaces, before they compromise structural reliability [6,33,90,100].

*Infrared thermography (IRT)*, through controlled excitation, can be used to estimate thermal diffusivity and conductivity, indirectly revealing structural damage, resin-rich zones, voids, or non-homogeneities in sandwich cores or cured laminates. This technique is particularly effective for detecting changes associated with delamination or other damage in composite materials. By employing excitation methods such as pulsed, lock-in, or step-heating thermography, infrared thermography quantifies thermal properties with high precision, enabling the characterization of material homogeneity, resin consolidation, and core integrity in fiber-reinforced polymers, carbon-fiber-reinforced polymers, and hybrid sandwich structures. Advanced data processing techniques, including thermographic signal reconstruction (TSR), principal component analysis (PCA), and machine-learning-based anomaly detection, enhance the accuracy of property characterization by isolating subtle thermal gradients linked to matrix defects, porosity, or interfacial weaknesses. These capabilities allow infrared thermography to assess early-stage degradation, such as micro-voids or delaminations, which alter thermal conductivity and impact mechanical performance. In multi-material systems, such as fiber–metal laminates or adhesively bonded composites, infrared thermography’s non-contact, full-field imaging provides critical insights into anisotropic thermal behavior and interfacial bonding quality, supporting quality control and material verification [25,33,77,96].

*Capacitive sensing* is a highly sensitive technique for characterizing local dielectric property variations in polymeric, multilayered, and insulating systems. By capturing permittivity shifts associated with mechanical, thermal, or environmental changes, it provides insights into material heterogeneity, moisture content, aging, void formation, and interfacial degradation. In applications such as FRP–metal interfaces or sandwich cores, capacitive measurements can reveal subtle phenomena like resin-rich zones, adhesive thinning, or incipient delamination, often beyond the resolution of conventional NDT methods. The technique’s compatibility with embedded and flexible architectures supports its integration into real-time monitoring frameworks for long-term performance assessment [49,52].

For functionally graded materials or systems with thermal/electrical gradients, these characterization tools are essential for in-process verification or performance benchmarking.

### 3.4. Structural Monitoring and Health Tracking

Many NDE techniques are now being integrated into advanced structural health monitoring (SHM) systems, enabling the continuous, in situ, or on-demand evaluation of structural performance over time [33,87]. Unlike conventional periodic inspections, these SHM-integrated approaches aim to detect the onset, progression, and interaction of damage mechanisms under real-world service conditions [4]. By capturing structural responses such as stress wave emissions, impedance shifts, or dielectric changes, SHM-enabled NDE facilitates proactive risk mitigation, predictive maintenance, and extended service life of critical components [49,73]. These systems are especially valuable in complex or hybrid structures such as fiber-reinforced polymers, sandwich composites, and fiber–metal laminates, where hidden or evolving defects may not be detectable through isolated inspections [52]. The continuous flow of sensing data further supports data-centric asset management, digital twin integration, and the transition toward intelligent, condition-based maintenance strategies [20,80].

*Acoustic emission (AE), electromechanical impedance (EMI), and fiber optic sensors* are among the most widely adopted techniques for structural health monitoring in sectors such as aerospace, civil infrastructure, and wind energy. These sensors are used in both embedded and surface-mounted configurations to provide continuous, real-time tracking of damage evolution in composite and hybrid systems. Acoustic emission systems offer early warnings of damage progression by capturing stress waves emitted during active structural degradation under service conditions. Their ability to detect evolving damage makes them suitable for long-term SHM, particularly in environments where manual inspection is limited. Electromechanical impedance sensors, using piezoelectric transducers, are effective at capturing localized changes in structural impedance, reflecting early-stage stiffness or bonding degradation. Their high sensitivity makes them well-suited for detecting micro-level damage in joints, interfaces, and layered assemblies. Fiber optic sensors, including Fiber Bragg Gratings (FBGs) and distributed systems, enable full-field monitoring of strain, temperature, and dynamic deformation, particularly in complex geometries and large-scale infrastructures. Their durability and compatibility with digital twin platforms make them ideal for condition-based maintenance and performance optimization [29,33,73,92,101,102].

*Capacitive and resistive sensor arrays*, when embedded within laminates or bonded to structural surfaces, enable continuous, distributed structural health monitoring across large and geometrically complex systems. These sensors detect spatial and temporal variations in physical parameters such as dielectric permittivity, electrical resistance, or strain gradients, making them highly effective for tracking moisture ingress, interfacial delamination, adhesive degradation, and low-velocity impact events. Their fine spatial resolution allows for the real-time mapping of localized anomalies that may otherwise remain undetected in wide-area components. In applications involving sandwich panels, fiber-reinforced polymers, and hybrid material systems, capacitive and resistive sensing arrays provide a lightweight and scalable alternative to traditional point sensors. These arrays can be conformally integrated into the structural layup without compromising mechanical performance, making them particularly suited for smart composite skins, bonded joints, and large-area infrastructure. When connected to wireless or edge-processing platforms, they enable autonomous structural health monitoring, support predictive maintenance, and facilitate integration with digital twin environments [33,52].

*Wireless sensing platforms* are increasingly adopted for structural health monitoring of hard-to-access or large-scale structures such as wind turbine blades, bridge decks, and aerospace components. When integrated with smart capacitive or resistive sensor arrays, either embedded within laminates or surface-mounted, these systems enablethe distributed, autonomous detection of damage mechanisms including delamination, moisture ingress, thermal gradients, and impact events. Their wireless nature allows for real-time data transmission, reduced cabling, and improved scalability, especially in composite and sandwich structures where conventional wired monitoring systems may be impractical or intrusive [73,103]. While wireless platforms facilitate autonomous data acquisition, the true potential of structural health monitoring is realized when these sensor networks are paired with intelligent data interpretation tools. To this end, machine-learning-based signal interpretation is being increasingly used to process and extract actionable insights from complex sensor outputs such as those generated by infrared thermography, acoustic emission, electromechanical impedance, and distributed optical or electrical arrays. Machine learning algorithms, particularly deep learning, support vector machines (SVMs), and convolutional neural networks (CNNs), enable the automated damage classification, anomaly detection, and trend prediction in structural performance. This integration supports the transition from conventional NDT toward predictive, data-driven maintenance paradigms, and aligns with the broader goals of NDE 4.0 and digital twin frameworks for intelligent infrastructure management [21,26,33,80,103,104,105].

*Machine-learning-based signal interpretation* plays a transformative role in structural health monitoring by enabling the automated analysis of complex data streams generated by advanced NDE sensors. These data, often originating from distributed sensor networks deployed in hybrid or large-scale structures, require sophisticated algorithms to extract meaningful indicators of degradation. By applying techniques such as supervised classification, unsupervised clustering, and deep learning models, including convolutional and recurrent neural networks, structural health monitoring systems can uncover latent damage patterns, distinguish critical failure modes, and forecast defect progression. These capabilities support real-time condition assessment and predictive maintenance strategies, reducing reliance on periodic inspections and improving structural reliability. In multimaterial systems, machine learning excels at fusing heterogeneous sensor outputs, enhancing damage localization, reducing false positives, and enabling holistic structural diagnostics. As structural health monitoring platforms advance toward autonomy and intelligence, machine learning becomes essential in transforming raw sensor outputs into actionable insights, aligning with the vision of data-driven, self-adaptive infrastructure management [33,80,104,106,107,108].

Monitoring is especially crucial in hybrid materials, where damage can begin at material interfaces or propagate in complex ways due to mechanical or thermal mismatches.

### 3.5. Integrity Validation and Lifetime Prediction

Beyond detection and monitoring, advanced NDT/NDE approaches serve as foundational tools for life-cycle engineering and structural reliability assurance. Their role extends into quantifying residual strength, predicting remaining service life, and validating structural integrity under evolving operational demands [4,6]. By continuously tracking defect initiation, propagation rates, and load-dependent behavior, these methods support the development of data-informed maintenance strategies, risk-based inspection protocols, and performance-based design frameworks [33,80]. Modern applications demand more than binary flaw detection; they require quantitative assessments of structural capacity, degradation severity, and long-term performance trends [2]. These functions are especially critical in safety-critical sectors such as aerospace, energy, transportation, and civil infrastructure, where failure consequences are severe [73]. Furthermore, NDT/NDE contributes to certification and recertification processes, supporting regulatory compliance, structural health documentation, and end-of-life decisions [4]. As material systems become more complex, incorporating hybrid interfaces, multifunctional layers, or damage-tolerant architectures, integrity validation and prognostic evaluation are central to ensuring sustainable operation and reliable asset management [49,80].

Ultrasonic and acoustic emission-based fatigue diagnostics play a pivotal role in structural lifetime prediction by enabling the continuous tracking of crack initiation, growth rates, and localized energy release under cyclic or environmental loading. These techniques are particularly effective in evaluating damage accumulation in layered assemblies, bonded joints, and hybrid composites, where traditional stress-life models may fall short [4,73]. Acoustic emission systems offer real-time insights into micro-crack nucleation and coalescence, while advanced ultrasonic methods, including phased array and time-of-flight diffraction, support the high-resolution profiling of internal fatigue-related defects. The integration of such methods with fatigue models allows for quantitative assessments of residual strength and structural degradation kinetics, especially in aerospace, nuclear, and energy-critical components.

To enhance the interpretability and reliability of these systems, recent advances in the AI-driven analysis of sensor datasets, including thermal, acoustic, and electromechanical impedance signals, have enabled the detection of subtle degradation trends that precede macroscopic failure. These time-series datasets, when processed through machine learning algorithms, reveal patterns associated with accelerated aging, overload cycles, or environmental stressors, enabling proactive maintenance before critical failure occurs [33,80]. Furthermore, multimodal data fusion platforms that integrate complementary sensing modalities such as ultrasonic, capacitive, infrared thermography, and acoustic emission are now being deployed to generate holistic integrity profiles. These systems support high-confidence decision-making in safety-critical sectors by bridging the gap between traditional inspection and predictive modeling, forming the core of emerging prognostics and health management frameworks [33,73,80].

Table 4 provides a summary of NDT/NDE techniques categorized by their functional purpose. Function-based classification clarifies how NDT/NDE techniques contribute to various stages of structural assessment, from initial defect detection to real-time monitoring and life estimation. This approach helps align methods with engineering objectives, especially in complex or hybrid systems, where distinct inspection strategies are necessary for different layers or interfaces. In advanced composite materials, no single technique is sufficient; instead, function-specific selection or strategic combinations such as capacitive sensing for detection and electromechanical impedance for monitoring are essential for reliable, comprehensive evaluation. As modern materials increasingly integrate functionalities like self-sensing or energy absorption, the ability of a technique to detect, localize, characterize, monitor, and predict becomes critical. Emerging research suggests that data-driven integration and machine-learning-assisted interpretation will shape the future of function-oriented NDE, with multi-functional techniques like capacitive sensing, acoustic emission, electromechanical impedance, and AI-enhanced thermography playing key roles, particularly when integrated into intelligent structural health monitoring platforms.

## 4. Classification of NDT and NDE Techniques by Application Domain

In addition to classifying NDT/NDE methods by their underlying physical principles or functional objectives, it is essential to consider their practical deployment across diverse industrial and material domains. While classifications in principle and purpose provide a theoretical foundation, real-world implementation hinges on the specific context in which these techniques are applied. Each application domain, ranging from aerospace and civil infrastructure to manufacturing, energy systems, and emerging technologies, presents distinct constraints, material behaviors, inspection geometries, environmental conditions, and defect modalities. These factors influence not only the selection of inspection techniques but also their adaptation, integration with monitoring systems, and compatibility with advanced material architectures. Modern domains increasingly rely on multi-material systems such as fiber-reinforced polymers, sandwich structures, fiber–metal laminates, and functionally graded materials, which demand sophisticated, often multimodal, NDE frameworks. Therefore, the deployment of NDT/NDE methods must be informed by domain-specific requirements, especially in systems with high structural complexity and stringent service conditions.

This section categorizes and critically analyzes NDT/NDE techniques based on their application in five major domains: (1) aerospace and aviation, (2) civil and infrastructure systems, (3) power generation and energy, (4) manufacturing and process control, and (5) emerging and specialized applications. Emphasis is placed on the alignment of inspection strategies with material systems, defect types, and in-service reliability needs, highlighting the role of NDT/NDE in advancing data-driven, performance-oriented inspection paradigms.

### 4.1. Aerospace and Aviation

The aerospace sector has long spearheaded advancements in NDT/NDE, driven by rigorous airworthiness certification standards, the critical need for structural reliability, and the continuous pursuit of lightweight, high-performance designs [8,109,110]. Aircraft components such as fuselages, wings, stringer-stiffened skins, bonded joints, and control surfaces now widely incorporate advanced materials, including carbon-fiber-reinforced polymers, fiber–metal laminates, and lightweight sandwich composites. While these multi-material systems enable exceptional performance and weight reduction, they also introduce complex inspection challenges due to their anisotropic properties, multi-layered architectures, and failure modes such as delamination, matrix cracking, and interfacial debonding [62,101,111].

To ensure airworthiness across both manufacturing and in-service phases, the aerospace industry employs highly reliable, multi-tiered NDT/NDE frameworks. Volumetric techniques such as phased-array ultrasonic testing and time-of-flight diffraction are commonly combined with surface-sensitive methods like infrared thermography and shearography. Additionally, acoustic emission monitoring during load testing and operational service provides real-time insights into damage initiation and propagation, particularly in composite fuselages and bonded structures. Capacitive sensing techniques, notably coplanar capacitive arrays, are also gaining traction for detecting dielectric property variations associated with moisture ingress, adhesive degradation, and delamination in fiber–metal laminates and complex joints [7,8,52,101,112].

Beyond conventional inspections, the aerospace sector is increasingly integrating embedded sensing platforms and structural health monitoring systems into structural designs. Distributed fiber optic sensors, capacitive sensor networks, and smart piezoelectric arrays are deployed to continuously monitor strain fields, thermal anomalies, and damage evolution throughout the flight lifecycle. This shift supports the emerging certification-by-analysis paradigm, where real-time data collection complements traditional inspections to validate airworthiness [4,8,73].

Overall, aerospace NDT/NDE strategies are evolving toward data-driven, multimodal, and prognostic-capable frameworks, combining the reliability of established methods with the intelligence of embedded sensing to meet the future demands of autonomous aerial vehicles, lightweight hybrid aircraft, and next-generation space systems. In this context, a variety of NDT/NDE techniques have been tailored specifically for aerospace applications. The following subsections systematically review the primary methods deployed in the aerospace industry, highlighting their operational principles, strengths, limitations, and suitability for inspecting advanced composite, hybrid, and lightweight structural systems.

*Ultrasonic testing (UT)*, particularly advanced variants such as phased array ultrasonic testing and time-of-flight diffraction, plays a crucial role in aerospace applications for ensuring the integrity of carbon-fiber-reinforced polymers, fiber–metal laminates, and sandwich composite fuselage structures. These methods enable deep penetration and precise detection of critical flaws, including voids, kissing bonds, and impact-induced delaminations, which are essential for maintaining airworthiness and structural resilience [2,6,25,113,114]. In modern aerospace manufacturing, ultrasonic testing is increasingly combined with robotic and automated scanning systems to improve inspection repeatability, throughput, and defect traceability, particularly during the fabrication of large composite assemblies such as fuselage barrels and tail sections [19,33]. Automated phased array inspection further streamlines maintenance by reducing inspection times and supporting stringent lifecycle management and quality assurance requirements [4].

*Acoustic emission (AE)* monitoring is widely employed during load testing, fatigue cycling, and operational service to provide real-time insights into critical damage mechanisms such as fiber fracture, matrix cracking, delamination onset, and adhesive debonding, particularly in aerospace assemblies like bonded joints, composite skins, and stringer-stiffened panels. By capturing transient elastic or stress waves associated with micro-crack initiation and growth, acoustic emission enables early-stage damage detection before macroscopic flaws develop, significantly enhancing maintenance decision-making, fatigue model calibration, and overall risk mitigation [33,73,115,116]. In aerospace applications, acoustic emission monitoring is increasingly integrated into fatigue test campaigns, structural certification programs, and life-extension initiatives for aging aircraft fleets. Continuous acoustic emission tracking during simulated load cycles supports the refinement of fatigue prediction models, enabling more accurate remaining life estimations for composite fuselages, bonded repairs, and high-cycle fatigue-prone structures. Recent advancements also focus on embedding acoustic emission sensors into composite panels during manufacturing, facilitating in-service structural health monitoring without the need for retrofitting [4,101,115,116].

*Infrared thermography (IRT) and shearography* are non-contact, full-field, and rapid inspection techniques extensively utilized in aerospace applications for detecting disbonds, delaminations, and core crushing within honeycomb sandwich structures and composite assemblies. Infrared thermography measures thermal contrasts caused by subsurface defects disrupting heat flow, whereas shearography detects minute surface strain anomalies correlated with hidden flaws under mechanical or thermal loading [63,117,118]. In maintenance and operational contexts, these methods are particularly effective for the rapid evaluation of lightweight secondary structures such as elevators, rudders, fairings, and access panels, where disbonds, impact damage, or core degradation may compromise aerodynamic performance and structural integrity. IRT is favored for its ability to rapidly cover large surface areas, making it ideal for periodic maintenance inspections, while shearography excels in the high-sensitivity detection of barely visible impact damage in carbon-fiber-reinforced polymer components, critical for damage-tolerant aerospace design philosophies [33,73,118,119]. Recent advancements have integrated these techniques with automated scanning platforms and AI-assisted image processing algorithms, significantly improving defect localization accuracy, inspection repeatability, and the evaluation of complex, curved aerospace surfaces [33].

*Radiography and computed tomography (CT)* are essential volumetric inspection techniques extensively utilized in aerospace manufacturing and maintenance for evaluating complex geometries in high-value components such as engine parts, turbine blades, structural fittings, and additively manufactured structures. By utilizing X-rays or gamma rays, conventional radiography generates two-dimensional projection images that expose internal discontinuities, including porosity, inclusions, cracks, and misalignments [2,89,120,121]. Computed tomography extends these capabilities by reconstructing high-resolution, three-dimensional internal representations from multiple radiographic projections, enabling the detection of subsurface defects, fiber waviness, voids, and intricate interfacial anomalies. Computed tomography is particularly critical for thick-section composites, sandwich structures, and complex additively manufactured lattice architectures, where detailed volumetric analysis is required to ensure airworthiness and structural integrity. In aerospace, computed tomography is indispensable for the certification of flight-critical hardware, non-invasive defect validation, and material characterization at micron-scale precision [2,73,121,122]. However, the high operational costs, large system footprints, and scanning time requirements typically constrain computed tomography usage to laboratory or manufacturing environments, limiting its deployment for in-service inspections. Recent innovations, including high-energy computed tomography systems, inline computed tomography for real-time additively manufactured monitoring, and AI-accelerated reconstruction algorithms, are progressively expanding computed tomography’s applicability toward faster, more integrated aerospace inspection workflows [33,123].

*Capacitive sensing*, especially through coplanar capacitive sensors, is emerging as a lightweight, non-contact technique for evaluating dielectric discontinuities, moisture ingress, adhesive layer integrity, and delamination in layered or insulating aerospace materials. Unlike conventional electromagnetic-based NDT methods that depend on conductive pathways, capacitive sensing operates by detecting local variations in dielectric properties, making it exceptionally suited for inspecting non-conductive composites, bonded joints, fiber–metal laminates, and polymer–metal hybrid structures where traditional eddy current and ultrasonic methods face significant limitations [7,52,112]. In aerospace applications, coplanar capacitive sensor technologies offer distinct advantages for the non-invasive evaluation of fuselage sections, wing skins, leading-edge assemblies, and secondary flight structures, where complex layer interfaces or limited conductivity challenge other NDT approaches. Capacitive sensors’ ability to detect subsurface voids, adhesive debonding, and aging-related dielectric property changes without requiring couplants or direct surface contact enhances their suitability for both periodic inspections and continuous structural health monitoring [33,52]. Recent advancements, including flexible, printable capacitive sensor arrays, AI-enhanced signal interpretation, and integration into autonomous robotic platforms, are further expanding the feasibility of real-time monitoring across large, curved aerospace surfaces, supporting next-generation structural health monitoring strategies [33].

The aerospace sector is a leading adopter of embedded structural health monitoring, digital twin frameworks, and AI-supported diagnostics, particularly in the context of airworthiness compliance and life-extension programs. NDE is increasingly integrated with these advanced tools to enable real-time condition assessment, support certification-by-analysis paradigms, and ensure continuous airworthiness throughout a component’s lifecycle [20,33,101,122].

### 4.2. Civil Infrastructure and Structural Health Monitoring

Civil infrastructure presents a distinct set of NDT/NDE challenges, shaped by the massive scale of structures, constrained accessibility, long service lifespans, and cumulative exposure to environmental stressors such as temperature fluctuations, freeze–thaw cycles, corrosion, and fatigue. Traditional material systems, including reinforced concrete, prestressed steel, and masonry, have been supplemented by modern advancements such as fiber-reinforced polymer retrofits, hybrid composite bridges, and prefabricated modular sandwich panels designed for enhanced performance and sustainability. These evolving material systems introduce new inspection complexities, particularly at bonded interfaces, layered joints, and multi-material transitions, where degradation modes are less apparent. Civil infrastructure NDT/NDE must therefore balance between global monitoring strategies capable of assessing entire spans, decks, or towers and localized high-resolution methods for detecting hidden cracks, delamination, moisture ingress, or rebar corrosion. Furthermore, inspection strategies must often accommodate restricted site access, variable operational loads, and the need for real-time or minimally disruptive evaluations to support preventive maintenance without compromising service continuity. This sector is increasingly adopting distributed sensor networks, embedded health monitoring systems, and intelligent data fusion frameworks to move from periodic manual inspections toward predictive, data-driven asset management paradigms [33,73,124,125]. The subsequent sections review the primary NDT/NDE methods employed in civil infrastructure, emphasizing their ability to diagnose various damage and support the long-term resilience of complex, multi-material systems operating under diverse environmental and service demands.

*Acoustic emission (AE) and electromechanical impedance (EMI)* techniques are widely integrated into structural health monitoring systems for critical civil infrastructure such as bridges, tunnels, dams, and offshore platforms. These methods enable the real-time detection of active damage processes, including crack initiation and propagation, delamination in fiber-reinforced polymer wraps, interface degradation, corrosion-induced microcracking, and loss of prestress. Acoustic emission captures transient stress waves generated during damage events, making it particularly effective for monitoring prestressed concrete elements, cable-stayed bridges, and fiber-reinforced polymer retrofitted beams under live loading and environmental fluctuations, where traditional visual inspections often fail to identify hidden or evolving damage. Complementarily, electromechanical impedance techniques, leveraging piezoelectric sensors, track localized changes in structural stiffness and mechanical impedance with high sensitivity, enabling the early detection of subtle degradation phenomena without requiring external loading. The integration of acoustic emission and electromechanical impedance into intelligent structural health monitoring frameworks supports data-driven maintenance strategies and risk-informed asset management, offering a powerful approach for extending the service life of aging infrastructure networks [29,73,126,127].

*Infrared thermography (IRT) and Microwave Testing* are effective non-contact techniques that are increasingly employed in the assessment of civil infrastructure, particularly for detecting subsurface damage, insulation degradation, joint deterioration, and bond failures in layered construction systems. Infrared thermography operates by visualizing thermal gradients induced by defects such as voids, delaminations, moisture ingress, and thermal bridging anomalies, making it highly suitable for inspecting building façades, precast insulated elements, bridge decks, concrete panels, and sandwich-based insulation systems, including those used in offshore platforms. Pulsed and lock-in thermography variants further enhance defect detection by improving thermal contrast at deeper layers. Microwave Testing complements infrared thermography by enabling subsurface interrogation beyond surface-visible damage, offering sensitivity to variations in dielectric properties associated with trapped moisture, adhesive failure, or material aging. This capability is particularly advantageous for thick composite façades, retrofitted structures, and insulated offshore platforms where thermal-only methods may have limited penetration. Together, infrared and microwave approaches provide non-destructive, rapid, and scalable solutions critical for condition-based maintenance, building exterior monitoring, and insulation integrity verification in modern and aging civil infrastructure [4,33,73,91,128,129]

*Capacitive sensing arrays* are emerging as an innovative and highly sensitive non-invasive technique for detecting dielectric property variations associated with moisture ingress, interfacial delamination, adhesive degradation, and void formation in civil infrastructure materials, such as fiber-reinforced polymer-strengthened concrete, sandwich composites, and modular wall systems. These sensors operate by detecting local changes in permittivity and are particularly effective for identifying early-stage deterioration long before visible cracking or spalling occurs. The scalability of coplanar capacitive sensor designs enables wide-area deployment across large bridge decks, tunnel linings, and layered structural walls, offering distributed sensing capabilities beyond those achievable with traditional spot-sensor methods. Furthermore, the ease of integration with embedded structural health monitoring platforms, including wireless data acquisition systems, positions capacitive arrays as a promising solution for continuous, autonomous condition monitoring in aging civil infrastructure. This approach significantly enhances predictive maintenance strategies by enabling the early detection of hidden damage mechanisms critical for service life extension [33,45,52].

*Embedded sensor networks*, fueled by the emergence of smart infrastructure and the Infrastructure 4.0 paradigm, are revolutionizing the deployment of NDT/NDE in civil engineering. These systems directly incorporate dense arrays of sensors, including fiber-optic, piezoelectric, capacitive, and resistive elements into structural components such as bridge decks, tunnel linings, and precast panels to enable real-time, autonomous condition monitoring. Embedded sensor networks transform static, point-in-time inspections into dynamic, life-cycle-based monitoring frameworks by continuously collecting and transmitting high-resolution data on strain, displacement, moisture content, and material degradation. Integrated with AI-enhanced analytics and cloud-based platforms, these networks not only support advanced functionalities such as anomaly detection, predictive maintenance scheduling, automated inspection planning, and risk-informed decision-making but also significantly improve the lifecycle management of civil assets. The seamless embedding of NDE capabilities within civil infrastructure aligns with smart city initiatives and resilient infrastructure strategies, offering a critical pathway for enhancing the durability, safety, and sustainability of next-generation transportation and utility systems [4,19,74,125,130,131].

### 4.3. Power Generation and Energy Systems

The energy sector, including nuclear, wind, hydroelectric, and oil and gas industries, demands robust and highly reliable NDT/NDE techniques capable of operating under extreme service environments characterized by high pressures, cyclic thermal loads, radiation fields, corrosive chemicals, and buried or submerged conditions. Critical components such as pipelines, turbine blades, pressure vessels, reactor internals, composite wind blades, and subsea installations require early-stage defect detection and continuous integrity monitoring to ensure operational safety, prevent catastrophic failures, minimize downtime, and extend operational life across diverse and often harsh applications [4,33]. Beyond traditional flaw detection, energy applications increasingly emphasize predictive degradation tracking, fatigue crack growth assessment, and corrosion mapping under service loads. The adoption of advanced materials such as composite materials, fiber-reinforced polymers, and hybrid laminates, particularly in turbine blades, offshore structures, and insulation systems, introduces complex damage mechanisms such as interfacial debonding and moisture ingress that demand multimodal NDE strategies [19,73]. To meet these evolving challenges, inspection solutions must not only deliver high sensitivity and depth penetration but also offer adaptability to automated robotic platforms, remote deployment, and integration with digital twin models and predictive maintenance frameworks. The growing reliance on structural health monitoring systems, equipped with wireless sensor networks, acoustic emission arrays, and fiber-optic sensing technologies, further highlights the sector’s shift toward intelligent, continuous asset management rather than periodic manual inspections [20,33]. The following subsections review the principal NDT/NDE techniques deployed in energy systems, with particular emphasis on their role in detecting degradation, supporting lifetime extension strategies, and ensuring the safe, efficient operation of complex material assemblies subjected to aggressive service conditions.

*Guided wave ultrasonics (GWUT), time-of-flight diffraction (TOFD), and phased array ultrasonic testing (PAUT)* are extensively employed for the inspection of long pipelines, weld zones, and steam generator tubes across the energy sector, including oil and gas transmission networks, nuclear plants, and offshore platforms. Guided wave ultrasonics are advantageous for screening large sections of piping from a single access point, offering long-range coverage ideal for detecting corrosion under insulation, weld defects, and anomalies in buried or coated infrastructure without extensive surface preparation. Time-of-flight diffraction provides high-resolution defect characterization and accurate sizing of internal flaws, making it highly effective for thick-walled pressure components and welded joints. Meanwhile, phased array ultrasonic testing enables advanced beam steering, electronic scanning, and sectorial coverage, allowing precise mapping of corrosion pits, pitting, laminate disbonds, and multilayer delamination commonly found in coated or composite-reinforced steel systems. Together, these ultrasonic-based techniques form a complementary inspection framework critical for early degradation detection, unplanned shutdown prevention, and the overall support of integrity management programs under demanding operational environments [2,4,33,73,132].

*Acoustic emission (AE)* monitoring plays a critical role in the structural health management of wind turbine blades, composite nacelle housings, and other rotating machinery subjected to dynamic, cyclic loads. By capturing transient stress waves generated during micro-crack initiation, fiber breakage, delamination propagation, or adhesive interface failure, acoustic emission enables the real-time detection of progressive damage mechanisms that can evolve under fluctuating wind loads, thermal cycling, and environmental exposure. Its in-service deployment provides embedded, volumetric monitoring capabilities, allowing the early identification of subsurface degradation, such as internal cracking, delamination, adhesive failure, and fatigue-related damage, before visible signs emerge. Unlike conventional surface inspections, acoustic emission supports condition-based maintenance strategies, minimizing unexpected downtime and extending operational lifespans through targeted repairs rather than costly full-blade replacements. Furthermore, the integration of advanced acoustic emission clustering and localization algorithms facilitates the accurate identification and mapping of damage-prone regions, enabling operators to distinguish between harmless acoustic phenomena, such as surface friction, and serious structural faults, an essential capability for maintaining the reliability of large-scale offshore and onshore wind farms [33,73,133,134].

*Fiber optic sensors* are increasingly embedded into large fiber-reinforced polymer wind turbine blades, composite nacelle structures, and pressure vessels to enable distributed structural health monitoring under operational and environmental stresses. Techniques such as Fiber Bragg Grating sensors, optical time-domain reflectometry (OTDR), and distributed strain and temperature sensing (DSTS) allow continuous tracking of strain fields, moisture intrusion, thermal gradients, and interfacial separation across vast surfaces and complex geometries. These systems enable the early detection of localized anomalies, environmental degradation, layer separation, and material fatigue, supporting proactive maintenance strategies and significantly reducing the risk of catastrophic blade failures. In addition to offering immunity to electromagnetic interference, lightweight integration, and the capability to multiplex hundreds of sensing points along a single fiber, fiber optic networks provide exceptional advantages for wind energy and offshore energy infrastructure. Their compatibility with composite manufacturing processes, such as resin infusion and pre-preg layup, further facilitates seamless in situ integration during blade fabrication, paving the way for smart composite structures capable of autonomous damage detection and real-time operational diagnostics [63,73,102,135].

*Microwave and thermographic techniques* are increasingly employed for the inspection of multilayer protective coatings, thermal barriers, and insulation systems in pipelines, pressure vessels, and aboveground storage tanks across the energy sector. Microwave-based sensing, utilizing sweep-frequency antennas or synthetic aperture radar configurations, enables the non-invasive detection of subsurface anomalies such as delamination, water ingress, disbonded layers, and corrosion under insulation (CUI) without requiring removal of protective cladding. Complementarily, thermographic techniques, including pulsed and lock-in infrared thermography, provide rapid, full-field thermal imaging to reveal insulation degradation, moisture saturation zones, and interfacial defects through subtle temperature variations. Their non-contact operation, rapid scan times, and scalability make both methods highly effective for large-scale inspections of aging oil and gas infrastructure, petrochemical facilities, and wind turbine towers, where traditional invasive techniques are costly and time-consuming. Together, microwave and thermographic approaches offer a powerful dual-modality solution for assessing the integrity of protective systems, enabling proactive maintenance planning and significantly extending the operational life of assets exposed to corrosive and high-temperature environments [33,73,136,137].

Due to increasing automation and safety demands, robotic NDE systems, IoT-enabled sensors, and AI-enhanced analytics are becoming integral to the energy sector. These technologies enable real-time reliability assessments, predictive maintenance, lifetime extension, and safe shutdown planning, ensuring optimized performance and safety across critical infrastructure [19,20].

### 4.4. Manufacturing and Process Control

Modern manufacturing, particularly in the production of advanced composites, hybrid joints, and additively manufactured components, increasingly relies on in-line and in-process NDT and NDE techniques for real-time quality assurance, defect detection, and property verification. As material architectures become more complex, integrating fiber-reinforced polymers, fiber–metal laminates, and multifunctional graded layers, traditional post-manufacturing inspections are often insufficient to meet the stringent demands for consistency, precision, and performance. This has created a growing demand for non-contact, rapid, and automated inspection methodologies capable of operating during critical manufacturing stages such as layup, curing, resin infusion, or additive layer deposition. Techniques such as infrared thermography, ultrasonic spectroscopy, shearography, and capacitive sensing are increasingly integrated into closed-loop manufacturing systems, enabling real-time defect detection, fiber orientation validation, void content monitoring, and interfacial quality assessment. In additive manufacturing, in situ monitoring methods, including computed tomography, acoustic emission, and electromechanical impedance techniques, are employed to detect volumetric anomalies such as porosity, incomplete fusion, and layer delamination, preventing defect accumulation. The convergence of advanced sensing technologies with Industry 4.0 paradigms, leveraging machine learning algorithms, digital twins, and predictive analytics, supports adaptive process control, dynamic defect correction, and enhanced process certification, particularly across high-performance sectors such as aerospace, automotive, and biomedical device manufacturing [2,4,19,33,73,80].

*Infrared thermography (IRT) and shearography* are increasingly deployed for the in-line and post-cure inspection of high-performance aerospace composite components, providing rapid, non-contact detection of critical subsurface anomalies. Infrared thermography captures localized thermal gradients during curing or cooling phases to identify voids, delaminations, and resin-starved or resin-rich zones without interrupting production, making it particularly valuable for large-area inspections in automated composite manufacturing lines [33,63]. Shearography, based on laser speckle interferometry, complements infrared thermography by detecting strain anomalies and internal defects such as disbonds, core crushes, and impact damage induced by thermal, vacuum, or mechanical loading. Its high sensitivity is especially advantageous for inspecting thin-walled and sandwich structures where hidden defects can critically impact structural performance and certification standards [73,81]. Together, these optical techniques provide fast, scalable, and Industry 4.0-aligned quality assurance solutions, enhancing precision and reliability in aerospace composite manufacturing.

*Ultrasonic spectroscopy* is increasingly employed for quality grading, resin infusion monitoring, and material verification during the manufacturing of advanced composites and hybrid laminates. Through the analysis of frequency-dependent acoustic characteristics like velocity, attenuation, and dispersion, ultrasonic spectroscopy allows for the accurate assessment of fiber orientation, matrix consolidation, and post-cure quality, especially in thick laminates and sandwich structures [3,6,138]. This technique supports the early-stage detection of voids, dry spots, and fiber misalignment, which are critical for ensuring mechanical performance and preventing downstream failures. Additionally, *electromechanical impedance (EMI)* methods are utilized to assess joint integrity and detect thermal degradation in hybrid metal-composite parts. By monitoring localized mechanical impedance variations through surface-mounted piezoelectric sensors, electromechanical impedance enables the in situ identification of weak bonding, thermal aging effects, or interfacial disbonds immediately after manufacturing processes such as curing, welding, or additive joining [33,88]. Together, these acoustic and electromechanical sensing approaches offer non-destructive, high-sensitivity alternatives to traditional destructive validation techniques, advancing intelligent process control and enhancing manufacturing reliability.

In additive manufacturing, especially for metal and polymer-based processes, advanced NDE techniques are critical for ensuring build integrity and minimizing defect propagation. Methods such as *computed tomography (CT)*, *Electrical Resistance Tomography (ERT)*, acoustic imaging, and infrared thermography (IRT) are increasingly deployed for in situ and post-process inspection. Computed tomography offers high-resolution volumetric imaging to detect internal porosity, incomplete bonding, lack of fusion, and microstructural anomalies in complex additive manufacturing parts [33,89]. Electrical Resistance Tomography enables the real-time monitoring of conductivity variations during layer deposition, revealing material anisotropy and early-stage defect formation without interrupting the build process [88]. Infrared thermography facilitates thermal field mapping and defect localization, identifying void formation, delamination, and residual stresses as the part is manufactured layer-by-layer [33,91]. These complementary techniques form the foundation of intelligent, closed-loop quality assurance systems in additive manufacturing, paving the way for autonomous defect detection, adaptive process control, and enhanced certification pathways for next-generation components.

With the increasing adoption of Industry 4.0 principles, NDE systems are rapidly evolving from traditional quality control tools into active components of smart manufacturing ecosystems. Advanced digital NDE platforms are now integrated with robotic manufacturing cells, additive manufacturing lines, and adaptive process control systems, enabling real-time sensing, dynamic defect detection, and closed-loop production management [19,33]. By linking outputs from techniques such as computed tomography, infrared thermography, and microwave inspection directly to manufacturing execution systems and adaptive control algorithms, these platforms allow immediate correction of production parameters, material feed rates, and thermal profiles. This transition toward closed-loop control drives the realization of zero-defect manufacturing, minimizes rework and scrap rates, and enhances production efficiency, particularly in high-value sectors like aerospace, biomedical device fabrication, and energy systems [20,80].

### 4.5. Emerging and Specialized Applications

Emerging applications of NDT/NDE span fields such as biomedical devices, additive manufacturing, space structures, and smart infrastructure. These sectors increasingly demand non-invasive, biocompatible, or extremely lightweight methods, often requiring customized or hybrid inspection strategies and the integration of sensors within the structure itself.

*Space applications* utilize ultra-lightweight materials such as carbon fiber reinforced polymers (CFRPs), sandwich panels, and metallic-ceramic hybrids, requiring low-mass sensors like coplanar capacitive sensors, electromechanical impedance, and Fiber Bragg Grating for launch and orbital inspection. These sensors, along with shearography, provide effective, low-weight inspection platforms for thin-walled carbon fiber reinforced polymers, honeycomb cores, and hybrid joints in space structures [7,73].

*Biomedical implants and composites* are an emerging and critical field for the application of NDT/NDE methods. Biomedical implants and composites, including implantable materials and prosthetics, are assessed using low-energy ultrasonic testing, infrared thermography, and emerging terahertz imaging, offering biocompatible, non-damaging evaluation with high sensitivity for detecting surface and internal defects [74]. Additionally, infrared thermography is being explored for medical diagnostics such as breast cancer screening and tumor detection, leveraging its ability to detect thermal anomalies linked to abnormal blood perfusion [139].

*Additive manufacturing* presents unique challenges for NDT/NDE due to its inherent geometric complexity, rapid cooling rates, and process-induced defects such as anisotropy, porosity, lack of fusion, microcracks, and layer misalignment. Conventional inspection methods often fall short in detecting internal flaws within dense or intricately curved additive manufacturing parts. To address these limitations, advanced techniques such as high-frequency X-ray computed tomography, laser ultrasonics, and machine-learning-enhanced thermography are increasingly employed for both real-time and post-process evaluation, particularly in aerospace-grade metals and polymers [21,33,80]. Furthermore, in situ monitoring systems utilizing acoustic emission or optical sensing are being integrated into additive manufacturing platforms to enable layer-wise defect detection and improved process control.

*Smart infrastructure and emerging soft systems*, such as soft robotics and wearable electronics, are increasingly incorporating embedded NDT/NDE sensors to enable autonomous, real-time structural monitoring. In infrastructure applications, technologies like radio-frequency identification (RFID), microelectromechanical systems (MEMS), and fiber optics are integrated with AI-driven diagnostic tools to support long-term monitoring, structural digitalization, and predictive design feedback for modular fiber-reinforced polymer-intensive systems [20,33]. Similarly, in the context of soft robotics and biomedical devices, flexible and stretchable sensors, including strain gauges, capacitive arrays, and self-healing sensor networks, are being developed to monitor strain localization, delamination, and functional integrity in highly deformable systems. Although still in the early stages, these innovations point to a future of embedded, adaptive, and autonomous NDE across a wide range of smart materials and applications [140].

These specialized domains are propelling advancements in hybrid, low-profile, and adaptive NDE technologies, encompassing multiphysics sensing, miniaturized NDT systems, and multimodal platforms. Many of these advancements leverage progress in material science, electronics, and edge computing and are tailored to lightweight, multifunctional, or biologically sensitive systems [3,110,141].

Table 5 provides a summary of NDT/NDE techniques categorized by their application domain. This classification of NDT/NDE techniques underscores the importance of contextual relevance in technique selection, especially for hybrid structures, advanced composites, and multi-layered systems. Each sector imposes distinct constraints from weight savings in aerospace to environmental exposure in civil infrastructure that require tailored, often hybridized inspection strategies. Across various sectors, technologies such as capacitive sensing, acoustic emission, electro-mechanical impedance, ultrasonic testing, and AI-integrated multimodal systems are increasingly recognized as key enablers. Their development is closely tied to the evolution of NDE 4.0, emphasizing integration with embedded architectures, automation, and predictive analytics.

## 5. Emerging Trends and Future Directions in NDT/NDE

Recent advancements in non-destructive testing and evaluation (NDT/NDE) mark a clear shift from periodic, manual inspections toward automated, intelligent, and continuous monitoring systems. This transformation is driven by progress in hybrid inspection platforms, embedded sensing technologies, and AI-enhanced analytics, which are redefining how structural integrity is assessed across complex, layered, and multifunctional materials. Modern multimodal systems now integrate ultrasonic testing, infrared thermography, acoustic emission, and capacitive sensors to capture complementary physical responses, improving the detection of critical defects such as delamination, interfacial degradation, and microcracking in composite structures [33,52,73]. Real-time, autonomous platforms leveraging active thermal excitation, digital shearography, and deep-learning-based image recognition enable rapid, full-field evaluation of large and geometrically intricate components [74].

The Total Focusing Method (TFM) and its extensions, such as Rayleigh-wave-based TFM and virtual source configurations, represent significant advancements in ultrasonic NDE. These approaches support real-time, high-resolution inspection of complex geometries, thick structures, and anisotropic materials, addressing limitations of conventional phased array techniques. With the ongoing development of post-processing algorithms (e.g., PCI, DMAS) and adaptive reconstruction modes, TFM is expected to play a central role in automated, high-fidelity structural health monitoring systems. Its compatibility with Industry 4.0 and digital twin architectures also makes it a cornerstone for future intelligent inspection platforms [64,65,68].

Simultaneously, the fusion of embedded smart sensors such as coplanar capacitive arrays, fiber optics, and printable electronics, with image-based diagnostics, is enabling scalable structural health monitoring architectures for curved and deformable surfaces [73,80]. These systems are increasingly deployed via edge-connected wireless networks and robotic platforms, extending NDT capabilities into hazardous or hard-to-access environments [19]. Embedded sensor networks using fiber optic lines, piezoelectric arrays, and flexible capacitive sheets are now integrated directly into the structural layup of composite and hybrid components, allowing real-time, autonomous condition monitoring with minimal disruption and enhanced insight into fatigue, impact damage, and moisture ingress.

The integration of machine learning, including convolutional neural networks, is improving the interpretation of ultrasonic, thermographic, and radiographic data, enabling predictive defect classification, adaptive inspection planning, and synchronization with digital twins [20,80,142]. In line with the Industry 4.0 paradigm, NDE 4.0 incorporates real-time monitoring, closed-loop feedback, and IoT-enabled communication to support predictive maintenance, reduce human error, and ensure continuous manufacturing quality. Unified calibration models and wave-based simulation frameworks are also being developed to enhance quantitative defect sizing and lifecycle assessment. Collectively, these innovations signal the emergence of NDE 4.0, an intelligent, interconnected, and autonomous paradigm in structural evaluation [19,21]. Table 6 presents a summary of emerging trends and future directions in NDT/NDE.

## 6. Comparative Evaluation and Selection Criteria

As NDT/NDE technologies evolve in parallel with increasingly complex materials and structural systems, the need for a systematic framework to evaluate and select appropriate techniques becomes imperative. In practice, the choice of an inspection method is influenced by several factors: not only the underlying physical principle but also the functional purpose, target material characteristics, defect types, and inspection context. Although advanced NDT/NDE methods offer high performance, no single technique proves effective across all applications. Their effectiveness varies significantly depending on material anisotropy, structural geometry, access constraints, service environment, and inspection phase during manufacturing, service life, or after damage occurrence. This section discusses key evaluation parameters that govern the selection process and presents a comparative assessment of widely used NDT/NDE techniques, particularly in the context of hybrid and layered materials.

### 6.1. Evaluation Criteria for NDT/NDE Selection

Selecting the most suitable NDT/NDE technique involves multiple performance criteria. Among the most critical are detection sensitivity, penetration depth, spatial resolution, material compatibility, and inspection adaptability. Detection sensitivity reflects the minimum defect size or damage signature a method can detect reliably, whereas penetration depth determines how deeply the technique can interrogate beneath the surface or through layers. For instance, ultrasonic testing offers significant depth penetration but can suffer from signal attenuation in porous or layered composites. Spatial resolution is particularly important when mapping delamination fronts, bond lines, or microstructural discontinuities, which is why methods like thermography or digital image correlation are employed in surface-sensitive analyses. Material compatibility remains a critical factor. For instance, although Eddy Current Testing excels with metallic components, it fails to produce reliable results on non-conductive polymers or fiber-reinforced composites. The method’s ability to conform to complex geometries, portability for field inspections, and integration with automated or digital systems are additional deciding factors, particularly in civil infrastructure and aerospace applications, where real-time diagnostics and robotic deployment are becoming the norm [4,6,73].

Economic considerations such as equipment cost, operational complexity, and the level of operator expertise required also play a pivotal role, especially for large-scale deployments. Emerging criteria include the method’s compatibility with machine learning pipelines and digital twin environments. These aspects are becoming more important as structural integrity management transitions from isolated inspection events to continuous, data-driven monitoring strategies under NDE 4.0 paradigms [19,20,21]. Overall, no single parameter should dominate the selection process; instead, the method must be evaluated holistically based on the operational context and technical objectives.

### 6.2. Comparative Performance of Major NDT/NDE Techniques

Different NDT/NDE techniques exhibit varied strengths and limitations depending on application constraints and structural material types. Ultrasonic testing, including phased array and time-of-flight diffraction, remains a cornerstone of volumetric defect detection and thickness mapping. It performs particularly well in homogeneous and metallic systems but struggles with signal attenuation and mode conversion in highly anisotropic or layered composites [6,25]. Acoustic emission, in contrast, provides real-time information about damage evolution, such as matrix cracking or fiber breakage and is especially useful in structural health monitoring systems. However, acoustic emission’s reliance on transient signals and its susceptibility to noise often require complex filtering and pattern recognition techniques [4,33].

Infrared thermography offers a fast, non-contact means for detecting surface and subsurface flaws like disbonds, voids, and delaminations. It is particularly effective when large-area inspections are required, such as in civil facades or aircraft panels. Its effectiveness, however, is constrained by depth limitations and environmental sensitivity, especially under passive conditions [80,91]. Radiographic testing and computed tomography remain the most detailed imaging techniques, capable of visualizing complex internal structures in three dimensions. Computed tomography is especially valuable in the inspection of additively manufactured parts or aerospace engine components, although it remains limited by cost, equipment size, and the need for controlled environments [2].

Capacitive sensing, particularly through coplanar capacitive sensors, has demonstrated strong potential for hybrid and dielectric-rich systems such as fiber-reinforced polymers and fiber–metal laminates. It offers the non-contact evaluation of dielectric property changes due to moisture, debonding, or internal void areas where traditional electromagnetic methods are often ineffective [49,52]. Electromechanical impedance is another emerging method that excels in detecting stiffness variations and localized structural degradation in bonded joints and smart materials. Its high sensitivity and lightweight implementation make it suitable for embedded structural health monitoring networks, though it is limited in inspection range [33]. Finally, the growing role of AI and machine learning has begun to reshape the field. Convolutional neural networks (CNNs) and other deep learning models are being used to enhance pattern recognition, automate defect classification, and enable predictive analytics in multimodal datasets [20,80,142].

### 6.3. Selection Strategies for Hybrid and Complex Materials

Hybrid structures and advanced materials such as sandwich composites, fiber–metal laminates, and functionally graded materials demand inspection approaches that address their multilayered, anisotropic, and interface-dominated characteristics. These materials often combine dielectric and conductive constituents, making single-mode inspection insufficient. For example, combining ultrasonic testing with capacitive sensing enables the complementary interrogation of both metallic layers and polymer-based cores. Similarly, infrared thermography, when used alongside coplanar capacitive sensors, can detect both thermal anomalies and dielectric changes associated with delamination or adhesive failure [49,73].

In the context of structural health monitoring, embedded arrays of electromagnetic interference, acoustic emissions, or coplanar capacitive sensors are increasingly deployed to monitor degradation over time. These sensor networks provide real-time tracking of damage progression, which is particularly useful in environments where structural access is limited or defect initiation is unpredictable. In highly engineered systems such as aircraft fuselage joints or wind turbine blades, where stress concentrations and interfacial failure are common, data fusion models supported by machine learning can improve diagnostic resolution and guide maintenance strategies [19,20,33]. For materials with spatially varying properties, such as functionally graded materials or additively manufactured parts, adaptive inspection paths and dynamic signal interpretation are required to account for property gradients and non-uniform responses. In these cases, the integration of sensor data with physics-based models or digital twins enables predictive performance monitoring and supports life-extension decisions. Table 7 provides a summary of the comparative evaluation of major NDT/NDE techniques.

## 7. Challenges and Limitations of Current NDT/NDE Methods

Despite major technological advancements in non-destructive testing and evaluation, several persistent challenges limit their universal effectiveness in the context of complex, layered, or hybrid structural systems. These limitations arise from both technical constraints (e.g., resolution, penetration, material compatibility) and systemic factors (e.g., cost, data interpretation, standardization). As NDT/NDE continues to evolve toward intelligent, automated, and embedded platforms, it is essential to recognize the limitations that must be overcome to ensure widespread adoption and reliable performance.

### 7.1. Material Complexity and Anisotropy

Modern materials such as fiber-reinforced polymers, fiber–metal laminates, and functionally graded materials pose inherent difficulties for many conventional techniques. Their anisotropic nature, layered construction, and varying dielectric or acoustic properties introduce signal distortion, wave scattering, and energy attenuation that degrade accuracy. For instance, ultrasonic testing often struggles in multilayered composites due to reflections at interfaces and internal damping, while eddy current and magnetic techniques are ineffective on non-conductive layers [4,6,25]. Hybrid structures also produce complex defect morphologies (e.g., delamination combined with interfacial debonding) that require multiple sensing modalities for reliable detection.

### 7.2. Limited Depth Resolution and Surface Bias

Techniques like infrared thermography, shearography, and capacitive sensing are highly effective for surface or near-surface defects but provide limited depth penetration. This presents challenges in thick-section composites, sandwich structures, and bonded joints where damage may evolve internally before reaching the surface. Conversely, radiographic methods like X-ray and computed tomography offer deep penetration but often require controlled environments, long exposure times, and expensive infrastructure, limiting their use in field conditions [2,33,49,80]. For many large-scale structures, achieving full-volume inspection with sufficient spatial resolution remains a key limitation.

### 7.3. Environmental Sensitivity and Field Deployment

Environmental factors such as humidity, temperature fluctuations, surface roughness, and electromagnetic interference can strongly affect NDT/NDE accuracy, particularly for capacitive, acoustic emission, and electromechanical impedance methods. Surface conditions can distort wave signals, reduce capacitance sensitivity, or introduce noise that masks damage signatures. In civil infrastructure or offshore environments, robust sensor packaging, calibration protocols, and environmental shielding are essential but often lacking in standard practice [33,73]. Moreover, many high-performance techniques remain confined to laboratory settings, with limited portability, automation, or adaptability to complex geometries.

### 7.4. Data Overload and Interpretation Bottlenecks

As NDT/NDE evolves into high-resolution, multimodal, and real-time systems, data volume has increased exponentially. Techniques like phased array ultrasonic testing, thermographic imaging, and AI-enhanced NDE platforms produce large datasets that require specialized algorithms for signal processing, pattern recognition, and defect classification. However, these systems still rely on operator expertise or rule-based interpretation in many industries, introducing subjectivity and limiting repeatability [20,80,142]. Additionally, many machine learning models used for NDE lack standardized datasets, real-world validation, and robustness to noisy or partial inputs, hindering widespread industrial trust.

### 7.5. Standardization and Integration Gaps

There is currently a lack of universal standards and certification protocols for emerging NDE techniques, especially for AI-based diagnostics, embedded sensors, and hybrid inspection systems. This gap complicates regulatory approval, cross-sector adoption, and benchmarking of performance metrics. Furthermore, the integration of NDE with structural health monitoring, digital twins, and predictive maintenance platforms requires seamless data fusion and interoperability, which are still under development in many fields [19,33].

Table 8 presents a summary of the key challenges associated with NDT/NDE techniques. The challenges facing current NDT/NDE techniques are multifaceted, ranging from fundamental physical limitations to emerging complexities in data and system integration. Addressing these issues requires not only the development of more robust, multimodal, and AI-integrated technologies but also advancements in standardization, materials science, and sensor design. For hybrid and multifunctional structures in particular, overcoming limitations related to anisotropy, depth sensitivity, and environmental stability will be crucial to enabling comprehensive, real-time, and intelligent inspection strategies.

## 8. Conclusions

Non-destructive testing and evaluation play an indispensable role in the assurance of structural integrity, safety, and performance across critical industries such as aerospace, civil infrastructure, energy systems, and advanced manufacturing. The continuous development of materials from traditional metals to complex hybrid composites and functionally graded systems has not only expanded the range of inspection needs but also redefined the expectations for NDT/NDE methods. This review has presented a comprehensive classification of NDT/NDE techniques based on their physical principles, functional purposes, and application domains, offering a structured framework to guide technique selection and integration, especially for emerging material systems.

As hybrid and multi-material structures become increasingly prevalent, the limitations of single-modality approaches have become more evident. Conventional methods such as ultrasonic testing, thermography, and radiography remain central to volumetric and surface inspections but often require supplementation with more sensitive or material-specific techniques such as capacitive sensing, electromechanical impedance (EMI), or acoustic emission (AE) to address layered, anisotropic, or dielectric-rich systems. The integration of smart embedded sensors, real-time monitoring platforms, and machine-learning-enhanced interpretation represents a major paradigm shift toward intelligent, predictive, and automated NDE, often framed under the umbrella of “NDE 4.0.”

Despite these advancements, several challenges remain, including signal degradation in complex materials, limited depth resolution in non-contact methods, and the lack of unified standards for AI-supported or embedded NDE systems. Addressing these barriers will require interdisciplinary collaboration across materials science, sensor design, computational intelligence, and industrial standards development. Additionally, the validation of machine learning algorithms, the scalability of embedded sensor networks, and the implementation of digital twins will be key enablers for next-generation NDE.

In summary, the future of NDT/NDE lies in the fusion of multimodal sensing, intelligent analytics, and adaptive deployment strategies. By embracing hybrid inspection platforms and developing robust, context-aware methodologies, the field is poised to meet the demands of increasingly sophisticated materials and high-performance structural systems, enabling safer, smarter, and more resilient engineering infrastructure.

While this review is designed as a comprehensive reference covering NDT/NDE techniques across materials, functions, and domains, it also reveals several areas warranting more focused investigation. These include emerging directions such as AI-enhanced diagnostics, capacitive sensing in layered dielectric systems, embedded structural health monitoring networks, and domain-specific strategies for composite aerospace or civil infrastructure components. Future review efforts may benefit from deeper analysis of these targeted subdomains.

## Figures and Tables

**Table 1:** Key advantages and limitations of non-destructive testing in comparison to destructive testing methods [9].

Non-Destructive Tests	Destructive Tests
**Advantages**	**Limitations**
1. All tests are performed directly on	1. Tests are not performed on the specimen
the actual specimens, enabling 100 %	directly. Therefore, the correlation
experimental evaluation of the real	between the sample and the object
components.	needs to be proven.
2. Many NDT techniques can be used	2. A single experiment may measure
on the same part, so many or all of the	only one or more characteristics.
features of interest can be measured.	
3. In-service testing is possible.	3. In-service testing is not possible.
4. Frequent inspections over a period	4. Measuring properties over a
of time are possible.	cumulative period of time cannot
	easily be possible.
5. A slight preparation of the	5. Specimen preparation is costly.
specimen is sufficient.	
6. Most NDT techniques are quick.	6. In general, high time requirements
	are needed.
**Limitations**	**Advantages**
1. Measurements are indirect reliability	1. Measurements are direct and
is to be verified.	reliable.
2. Usually, measurements are qualitative	2. Usually, measurements are
and can also be conducted quantitatively.	quantitative.
3. Experience and skilled judgment are	3. Correlation between measurements
required to interpret the measurements.	and material properties are direct.

**Table 2:** Key milestones in the evolution of non-destructive testing (NDT) technologies.

Period	Milestone/Development	Significance
Late 19th Century [9,11]	Discovery of X-rays (1895)	Foundation for radiographic testing
Early 20th Century	Development of magnetic	Enabled surface-level crack detection
[12,14]	particle testing and liquid	
	penetrant testing	
Post-World War II	Introduction of ultrasonic	Allowed internal flaw detection in
[12,15]	testing and eddy current	metallic and composite materials
	testing	
Late 20th Century	Digitization of radiography	Enhanced resolution, automation,
[11,13,15]	and ultrasonic phased arrays	and data processing
Early 21st Century	Integration of AI, IoT, digital	Enabled real-time diagnostics,
[18,19,20]	twins (NDE 4.0)	predictive maintenance, and
		continuous monitoring
Recent Advancements	Emergence of capacitive sensing	High-sensitivity diagnostics for
[33,34,44,49]	and multimodal inspection systems	complex materials; facilitates AI-
		enhanced SHM
Ongoing Innovations	Development of hybrid techniques	Reduces human error; improves
[16,19,22]	and automated robotic inspection	coverage, consistency, and
		accessibility in complex environments

**Table 3:** Summary of NDT/NDE techniques classified by physical principle.

Technique	Physical Principle	Typical Application	Material Compatibility	Contact Mode
Ultrasonic Testing	Mechanical	Internal flaw detection,	Metals, composites,	Contact
(UT, PAUT, TOFD)	(Elastic waves)	thickness measurement	laminates	
[2,6,25,59,63]				
Acoustic Emission	Mechanical	Real-time damage	Composites, hybrids	Passive
(AE) [4,6,29,33,73]	(Elastic waves)	monitoring		contact
Eddy Current Testing	Electromagnetic	Surface crack detection	Conductive metals,	Contact/
(ECT) [2,4,6,17]			FMLs	Proximity
Magnetic Particle	Electromagnetic	Surface defect detection	Ferromagnetic metals	Contact
Testing (MT) [1,2,14]		in		
		ferromagnetic steels		
Capacitive Sensing	Electromagnetic	Delamination, moisture,	Composites, hybrids,	Non-contact
(CCS) [34,49,52,76]		dielectric variation	dielectrics	
		ferromagnetic steels		
Microwave Testing	Electromagnetic	Adhesive joints,	Concrete, composites,	Contact/
[25,33,60,73,75]		thick composites	hybrids	Embedded
Infrared Thermography	Thermal/Optical	Subsurface voids,	Composites, FRPs,	Non-contact
(IRT) [4,6,25,63,79]		delamination	sandwich panels	
Shearography	Optical	Disbond detection,	Composites,	Non-contact
[54,73,81,82]		strain anomalies	honeycomb, hybrids	
Digital Image	Optical	Full-field strain	Composites, metals,	Non-contact
Correlation (DIC) [33,83,84]		mapping	hybrids	
Terahertz Imaging [25,33]	Optical/	Low-density	Polymers, multilayers	Non-contact
	EM hybrid	composite inspection		
Radiography (X-ray,	Radiographic	Volumetric defect	Metals, dense	Non-contact
Gamma) [1,2,6,85]	(Ionizing)	detection	composites	
Computed	Radiographic	High-res 3D imaging	Complex composites,	Non-contact
Tomography (CT) [2,25,89]	(3D imaging)	of defects	metals	
Neutron Radiography	Radiographic	Hydrogen detection,	Polymers, composites	Non-contact
[25,33,86]	(Neutron)	moisture, inclusions		
Electrical Impedance	Electrical	Moisture, cracks,	Metals, composites,	Contact
and ERT [4,33,87,88]		corrosion	hybrids	
Electromechanical	Electrical/	Local stiffness	Metals, composites,	Contact
Impedance (EMI) [33]	Mechanical	monitoring	SHM	
Hybrid/Multimodal	Multiphysics	Enhanced defect	All (tailored)	Mixed
Techniques [33,63,73]		detection		

**Table 4:** Summary of NDT/NDE techniques classified by functional purpose.

Functional Category	Technique	Primary Mechanism	Key Capabilities
**Defect Detection**	Ultrasonic Testing	Elastic wave propagation	Detects internal flaws,
	(UT, PAUT, TOFD)	monitoring	delamination, voids, even
	[4,6,25,63,90]		in curved/layered geometries
	Eddy Current Testing (ECT)	EM induction/Magnetic	Surface and near-surface flaw
	and MT [2,4,6,14,17]	leakage	detection in metals
	Radiographic Testing	Ionizing radiation/	Internal porosity and inclusion
	(RT), CT [1,2,25,85,89]	Magnetic leakage	detection with CT offering 3D
			reconstructions
	Infrared Thermography	Surface temperature contrast	Detects disbonds, voids,
	(IRT) [33,73,77,91]		moisture across large
			FRP/sandwich areas
	Capacitive Sensing	Dielectric permittivity	Detects delamination, moisture,
	[17,33,49,52]	variation	adhesive failure in insulating
			or dielectric layers
**Damage**	Acoustic Emission (AE)	Triangulation of transient	Real-time crack/delamination
**Localization**	[4,25,29,33,73,92]	elastic waves	localization under stress
	Shearography/DIC	Surface deformation mapping	Visualizes strain anomalies or
	[25,33,54,63,93,94,95]		damage progression under loading
	Infrared Thermography	Spatial heat flow anomalies	Localizes damage via thermal
	[25,33,77,80,96]		gradients and segmentation
	Microwave Testing	EM reflection variation	Detects/locates adhesive disbonds,
	[25,33,73,97,98]		voids, moisture in thick FRPs
**Property**	Electromechanical	Impedance variation due	Monitors local stiffness
**Characterization**	Impedance (EMI), EIT	to stiffness change	degradation, damping,
	[6,25,88,99]		or conductivity
	Ultrasonic Spectroscopy	Frequency-based material	Assesses modulus, bonding
	[3,6,25,33,90,100]	analysis	quality, matrix consolidation
	Infrared Thermography	Thermal diffusivity estimation	Estimates thermal conductivity;
	[25,33,77,96]		identifies resin-rich zones,
			delamination
**Structural**	Acoustic Emission (AE),	Embedded/passive sensing	Real-time monitoring of fatigue,
**Monitoring**	EMI, Fiber Optics		delamination, crack growth
	[29,33,73,92,101,102]		in operation
	Capacitive and Resistive	Distributed dielectric/	Tracks delamination, moisture
	Sensor Arrays [33,52]	resistive sensing	ingress, impact zones in
			layered composites
	Wireless SHM	Remote embedded	Long-term monitoring of hard-
	Platforms [26,33,73,80,103,104,105]	smart sensing	to-access systems like turbines
			and bridges
**Integrity**	AE and UT fatigue tracking	Cyclic load-based damage	Supports fatigue life modeling,
**Assessment**	[4,73]	progression	particularly for bonded joints
			and laminates
	AI-enhanced EMI,	Signal pattern recognition	Tracks damage growth, enables
	AE, IRT [29,33,80]		failure forecasting from time-
			series trends
	Multimodal Fusion	Combined data streams	Provides high-confidence
	[33,73,80]	from multiple domains	diagnostics, integrity profiles, and
			structural certification

**Table 5:** Summary of NDT/NDE techniques classified by application domain.

Application	Primary Materials/	Key NDT/NDE	Main Purposes	Highlights for Hybrid/
**Domain**	**Challenges**	**Techniques**		**Composite Systems**
**Aerospace and**	CFRPs, FMLs, bonded	UT (PAUT,	Flaw detection,	Multimodal methods
**Aviation**	joints; fatigue,	TOFD), AE,	fatigue monitoring,	for depth/surface
	delamination, moisture	IRT, CT, CCS	certification	synergy
**Civil**	Concrete, steel,	AE, EMI, IRT,	Crack monitoring,	Embedded sensors
**Infrastructure and**	FRP wraps;	Microwave,	debonding, moisture	(EMI, CCS) and IRT
**SHM**	aging, large scale,	CCS	ingress	for real-time
	exposure			distributed SHM
**Power**	Metals, composites;	GWUT, PAUT,	Pipeline integrity,	Robotic and wireless
**Generation and**	corrosion, thermal	AE, IRT,	fatigue, corrosion	systems for remote,
**Energy**	cycling, buried	Fiber Optics		detection
	access			hybrid-layer
**Manufacturing**	Laminates, AM parts,	IRT, Shearography,	In-line defect	Real-time inspection
**and Process**	joints; porosity,	CT, EMI, UT	detection, material	of thick composites
**control**	bonding,		consistency, Quality	and hybrid AM layers
	quality control		control	
**Emerging and**	FGMs, biomedical,	CCS, EMI,	Light-weight sensing,	Adaptive sensors and
**Specialized**	smart and space	AI-enhanced NDE,	damage tracking,	AI for multifunctional and
	structures	IRT	automated diagnostics	layered smart materials

**Table 6:** Summary of emerging trends and future directions in NDT/NDE.

Trend/Technology	Description	Key Applications	Impact on Hybrid/
			**Advanced Structures**
**Smart Embedded**	Integration of CCS, EMI,	FRP bridges, wind blades,	Real-time monitoring of
**Sensors**	and fiber-optic sensors	aerospace panels	delamination, interfacial
[33,49,52]	directly into structures		failure, and moisture ingress
**Flexible & Printable**	Conformal, low-profile	Biomedical devices, soft	Facilitates embedded
**Electronics**	sensing platforms for	robotics, space structures	sensing in non-planar,
[73,80]	curved or soft surfaces		anisotropic, or biocompatible
			materials
**Multimodal NDE**	Fusion of UT, IRT,	Aerospace, infrastructure,	Enhances defect detectability
**Systems**	AE, CCS, microwave,	sandwich composites	across layers with varying
[7,33,73]	etc. into integrated		electromagnetic and acoustic
	platforms		properties
**Robotic & Edge-**	Autonomous and semi-	Wind turbines, pipelines,	Scalable inspection of
**Connected SHM**	autonomous deployment of	aircraft, inaccessible	large or remote systems;
**Platforms**	NDE tools with wireless	structures	supports continuous and
[19,20]	data transmission		adaptive monitoring
**NDE 4.0 & Digital**	Integration of NDE with	Digital manufacturing,	Enables closed-loop
**Twins**	for image classification,	asset lifecycle management	decision making, real-time
[19,20,21,33]	signal denoising, and		analytics, and predictive
	predictive maintenance		structural behavior modeling
**Advanced Material**	Tailored sensing for	Aerospace, biomedical,	Drives development of hybrid,
**Adaptation**	FGMs, FMLs, architectured and	structural composites	adaptive NDE tools that address
[21,33,80]	multifunctional materials		material heterogeneity and
	predictive maintenance		anisotropy

**Table 7:** Summary of comparative evaluation of major NDT/NDE techniques.

Technique	Key Strengths	Limitations	Ideal Application
			**Domains**
Ultrasonic Testing (UT)	Deep penetration,	Signal attenuation in	Metals, bonded joints,
incl. PAUT, TOFD	high resolution, suited	composites; coupling	thick laminates
[2,6,25]	for internal flaws	required	
Acoustic Emission (AE)	Real-time monitoring,	Requires interpretation;	SHM of FRPs, tendons,
[4,33]	detects active damage	noise sensitive	wind blades
	under load		
Infrared Thermography	Fast, non-contact,	Limited depth,	Sandwich panels,
(IRT) [73,91,143]	large-area inspection	dependent on thermal	FRP facades,
		contrast	debonding zones
Radiography/CT	High-resolution	Costly, radiation	Additive manufacturing,
[2,80]	internal imaging,	hazard, lab-restricted	aerospace engines
	3D defect analysis		
Electromechanical	Highly sensitive	Localized detection,	Bond lines, thermally
Impedance (EMI)	to local stiffness	calibration needed	aged joints, SHM
[3,33]	and bonding changes		systems
Capacitive Sensing	Effective on dielectric,	Sensitive to distance,	FRPs, FMLs, polymer-
[49,52]	layered, or hybrid	surface roughness	metal interfaces
	structures; non-contact		
AI/Machine	Enhances defect	Requires large,	Multimodal systems,
Learning-Assisted	classification, supports	labeled datasets;	automated inspection,
NDE [20,80,142]	predictive maintenance	validation critical	digital twins

**Table 8:** Summary of the comparative evaluation of major NDT/NDE techniques.

Challenge Category	Description	Implications for Hybrid/
		**Composite Systems**
Material Complexity and	Heterogeneous layers, anisotropic	Signal distortion, reduced accuracy
Anisotropy [4,6,25]	properties, interface reflections	in FMLs,
Depth Limitation/	Limited penetration for IRT,	Difficult to inspect thick laminates
Surface Bias [2,91,143]	shearography; internal damage	and internal defects in multi-layer
	may be missed	systems
Environmental Sensitivity	Effects from temperature, humidity,	Performance loss in civil/offshore
[33,73]	EMI, and rough surfaces on sensor	applications; need for ruggedized
	response	designs
Data Overload and	High-resolution systems generate	Multimodal fusion is complex; ML
Interpretation [20,80,142]	large datasets; requires AI or expert	models need robust training and
	interpretation	validation
Standardization and	Lack of unified protocols for emerging	Delays in certification, poor
Integration [19,33]	methods and AI-driven systems	interoperability, challenges in SHM
		/digital twin link

## Data Availability

Data sharing not applicable.
